# Supplementary material for: Quantification of disease-associated RNA tandem repeats by nanopore sensing
Source: Nat Commun. 2026 May 8;17:6219. doi: 10.1038/s41467-026-72819-5 (PMC13369952; doi:10.1038/s41467-026-72819-5)
Supplement: Supplementary file 3 — Transparent Peer Review file [file 41467_2026_72819_MOESM3_ESM.pdf]

nature portfolio

Peer Review File

Quantification of disease-associated RNA tandem repeats by nanopore sensing

Corresponding Author: Dr Filip Bošković

This file contains all reviewer reports in order by version, followed by all author rebuttals in order by version.

Version 0:

Reviewer comments:

Reviewer #1

(Remarks to the Author)
The study utilised an extensively tried and tested and characterised solid-state nanopore methodology from the research group for repeated expansion detection. From this perspective, the novelty of this paper must stem from design of strategy and how authors adopt this strategy for the proposed detection of repeat expansion. Based on this I have the following comments:
The system from publisher listed Boskovic, F. as first author, but in the pdf Patino-Guilllen, G. is the first author?
Authors referring the hybrid construct as RNA nanostructures but it is in fact a RNA:DNA hybrid nanostructures as the formation of said RNA nanostructures contains more DNA than RNA, since the DNA oligos are longer with an end sticking out for the biotin, this confused me as I thought you assembled the entire structures as a RNA:RNA duplex (dsRNA) structure.
Please provide a gel of the final RNA:DNA hybrid structures.
The author assumes that for a say 51 CUG (153 nt) repeats, there will be 5 x (CAG)10 (5 x 30 nt) attached to the nanostructure. This is assuming every DNA oligos are at the right place and desired position. Is the authors saying this is an absolute (either full 5 attached, or no attached) or is that more likely to be a distribution of possibilities? This changes how readers interpret the results. So far from the writing, a strong sense of absolution can be sensed. My humble opinion is that the system should fluctuate at least from 4 x (CAG)10 to 6 x (CAG)10
Constructions of the nanostructures, Figure S8 tested some ranges: 3 times excess, and then 9 times excess to find the range to fully cover the CUG expansion region, then the construction of the nanostructures utilise 5 times excess. Why the difference?
Additionally, do you mean 5 times excess on all oligos in all regions? In a sequence specific region like the region outside the repeat of 1 RNA molecule, you have a roughly 1 region (say 10 nt) to 5 oligos of 10 nt. For the expansion region of 1 RNA molecule, a 5 repeat region will take 5 oligos of 10 nt, in a folding excess of 5 fold excessive DNA oligos, the expansion region is not in excess. Which from Figure S8, you produced multiple isoforms.
I can see some reversible clogging, the events are sparse, can the authors indicate how long the assay on average will be run? 1 hour, 2 hours, 3 hours, overnight? To collect enough data for this assay, say 100 events including false positives.
Figure 1a, maybe clearly label which end is the 5'.
Figure 2, placement of letter d is confusing as it merges into panel 2b, the box label from 2b can be moved to a better position to avoid confusion.
2d utilises Welch's t-test, for multiple data comparison, an ANOVA should be used in multiple comparison to minimise Type-1 error.
With regards to transfection and extraction detection, transfection can cause different levels of expression of certain construct, thus the mRNA (GFP in this case) will and should be the dominant species in the RNA pool without need to further refine the RNA samples. I wonder if authors can provide some comments on how this can be translated into real scenario, e.g. the diagnostic of say DM1, i.e. you isolated some levels of total RNA from a patient or animal model, and you perform the nanopore with this assay, the transcript is probably not abundance in your total RNA pool, since the issue is not that the disease caused over expression of this mRNA?
The SI is quite a big document, I suggest having all the sequence as a separate pdf file or excel file (if allowed).
Data availability said is provided, but not clear on how are these being provided? Repository?
Overall, while unique in trying to tackle the problem of repeat expansion detection, there are some caveats remain to be addressed or acknowledged the limits.

Reviewer #2

(Remarks to the Author)
I'm excited to read a novel method for detecting short tandem repeat expansions. As noted but the authors, while long-read sequencing has been incredibly valuable in this field, the DNA input requirements and expense have hindered adoption into clinical labs. This method may provide the basis for a new approach to testing these loci.

Major:
Why did you choose DM1, DM2, and CCHS1? How well do you expect this technique to generalize to additional STR loci? For example, do you expect it to perform well on other motifs, especially those with more extreme GC content, e.g. FAME loci (20% GC) or C9orf72 (100% GC)? Would you expect it to work on VNTRs (7+ bp motifs)?
Does this technique allow you to directly estimate the allele size for each sample, or does it focus on distinguishing between size categories (pathogenic vs benign)? It appears that only one intermediate or pathogenic-sized allele was tested for each locus. If relevant, could you please elaborate on what would be needed to estimate allele sizes, including those much larger than those tested.
How would this method handle interruptions and motif variations?
"The tandem repeat size distributions agree with the spread observed for the repeat sizes of the initial DNA plasmids used for RNA synthesis." Could you please plot the distribution of (inferred) allele sizes observed for each experiment and visually compare to the expected size? The data presented is almost exclusively in "signal" space. This would be critical to assess the degree of stutter/mosaicism as well as estimating accuracy.
While code was provided (https://github.com/maxearle/nas2), no usage documentation was available. Sample input data would also be needed to test it.

Minor:
Line 34: This is a little out of date. I would put it at 65-68 STR loci are associated with mongenic conditions, depending on how you count. So it would be more accurate to say more than 60 and cite a more recent paper. Also, by my count, ~30 STR of those loci are coding. If I'm understanding correctly, this technique would only work on coding STRs, so this is the true number of loci that are in scope. You may consider adding the total number of coding STRs (not just pathogenic ones) to demonstrate the potential scope of your work. Or, could the method be adapted to DNA?
Line 40-41: "The severity and onset of REDs [typically] depend on the number of repeats". For some diseases, it affects onset, in some severity, and in others, both. Interruptions and motif play a major role.
Line 54: You should include PacBio sequencing (both WGS and PureTarget) in this background.
Line 70-71: "trinucleotide repeats" DM2 is a 4bp motif
Line 349: "our method paves the way for detecting interruptions". Could you please explain this point with regard to the experiments presented in this paper? How would the method be adapted to do this?

Reviewer #3

(Remarks to the Author)
This study uses present a single-molecule nanopore-based strategy that enables direct quantification of tandem repeats in native RNA,RNA nanostructures enabled detect and distinguish disease-associated repeats in DM1, DM2, and CCHS1.

This study has multiple major limitations:

In your paper, you describe the use of DNA oligonucleotides and tandem repeat–containing RNA to construct RNA nanostructures for copy number assessment. Your approach involves in vitro transcription from DNA followed by hybridization with complementary DNA oligonucleotides. Given the high diversity and complexity of tandem repeat sequences, you present only a few examples to illustrate the feasibility of the method, and the DNA oligonucleotides used in these experiments may not be broadly applicable. Could you elaborate on how you envision addressing the sequence diversity and throughput challenges in practical applications?

In Figure 2d and Figure 3b, you demonstrate that different copy numbers of the motif repeats, identified using RNA nanostructures, are associated with varying degrees of ionic current reduction. However, it is worth noting that even among RNAs with the same repeat copy number, the current depth exhibits considerable variability, raising doubts about its ability to precisely quantify changes in tandem repeat copy numbers.

First, on page 2, line 94, you define abnormal expansion of the CNBP gene in DM2 as greater than 57 CCTG repeats. However, according to the most recent literature, the pathogenic threshold is generally considered to exceed 75 repeats. Could you clarify whether selecting 57 repeats as the cutoff for abnormal expansion is appropriate? Furthermore, non-pathogenic CCTG expansions are often interrupted by GCTG or TCTG motifs. Your methodology does not appear to describe how such interruptions within the CCTG repeats region were identified or differentiated. In addition, could you elaborate on whether the insertion of GCTG or TCTG motifs affects the ionic current signals measured through nanopores, and how the magnitude of such reductions can be distinguished from those caused by CCTG repeats? Clarifying this would be important for accurately determining the true number of CCTG repeats.

On page 17, line 497, you state that plasmids containing 10, 18, and 34 CTG repeats were obtained and transfected.

However, in the subsequent analyses, current spikes corresponding to the 10 and 18 CTG repeats were not reported. It would be helpful if you could clarify whether these data were collected but not shown, or elaborate on the reasons for their absence. Including these results, if available, would provide a more complete understanding of the relationship between repeat copy number and current spike patterns.

Version 1:

Reviewer comments:

Reviewer #1

(Remarks to the Author)
The revised manuscript addressed concerns that I raised from the last round of review. Now the revised edition lead to some more questions for the author to clarify:
1. The newly added S36 of the gel structure shows two bands with one near 3 kbp and on closer to 1 kbp. Say around 1500 bp will give you approximately 990,000 g/mol. With the 100 kDa MWCO filter cut-off purification, it will remove oligos and retain both the fully hybridised RNA:DNA nanostructures and the prematurely terminated transcripts. SO both of these are the analytes since the author did not mention about further isolation of the correct fragment. It looks like based on density, it is almost equal, do you observe translocation events of both fully formed and truncated RNA:DNA nanostructure. If so what proportion, this determines the realistic potential of this tool to study the expansion related disease (e.g. if recording of 8 hours yield 100 events, and 60 of them are truncated form, then the actual yield of the assay is 8 hours for 40 events, it complicates the assay design). Same question with the mRNA detection, how often do you detect the RNA:DNA labelled construct? or do you only detect the construct?
2. Experiment hour ranges from 2 to 8 hours even. Since the molecules only goes into the pore under certain polarity of voltage (it goes to anode/nanopore/capillary) from the bath (cathode), under constant DC potential over significant period of time will introduce drift in the current baseline, which can affect the signal quality (e.g. spike depth, area...etc), can the author clarify this?
3. Also drying of the reservoir over time? Does the salt concentration slowly increases overtime here due to evaporation, the author did say it is almost fully sealed in PDMS, but that require further justification.
4. While multiple nanopore system could be used, the cost of equipment also goes up if I am not wrong, you will require increasing number of high spec electronics and headstages to record the signal? With the cited ref 54 on a 16 channel system, the spec is a lot lower and I do question whether the author can still resolve the signal trace and spike properly with a lower spec sampling rate of 20 kHz versus the sampling rate here at 1000 kHz (1 mega-Hz) which is 5 times faster, and would be important to see the events barcode (1111 1001...etc)
5. The mRNA detection, the case presented here is very distinctive of no CUG expansion then against a CUG expanded version of it. to claim it has usage for intracellular detection accurate expansion determination, it should be tested against say 28 CUG expansion versus 34 CUG expansion, then it will demonstrate the value of this new tool better.
Based on the information presented
6. The new Figure S16, it looks like the signal mean reach maximum at <89 CUG, do the author expect there will be a maximum spike signal drop in this system? i.e. the tool cannot distinguish 51 CUG and for argument's sake, 89 CUG? because the signal spike drop are the same?

Reviewer #2

(Remarks to the Author)
The authors have addressed the majority of my comments. The additions give me greater confidence in the generalizability and accuracy of their approach.

I have one remaining concern about code documentation. I checked the provided URL (https://github.com/maxearle/nas2). Indeed, a README and test data have been added in response to my previous comment; however, there is still insufficient information for me to easily test/use the code. It is possible that this URL is out of date (last edited 3 months ago); so please update it if that is the case.

The README should provide sufficient information for a completely new user to install and run the tool. Here is a resource that provides a good summary and a simple example: https://www.makeareadme.com/

To summarize, at a minimum, the main (top-level) README should include:
- A brief description of the project
- Install instructions (these are present in the current version, but not well formatted)
- A sample run command for the test data
- A list of relevant commands/subcommands/scripts, their uses, parameters and inputs/outputs, including any non-standard formats.

Reviewer #3

(Remarks to the Author)
The revision of the manuscript by Patino-Guillen and collaborators has addressed most of the concerns raised during the

initial review. However, the weak point of the study remains the accuracy of hybridization of RNA:DNA hybrid structures in practical applications. In each individual containing variable TR motifs and their corresponding RNA transcripts, the inherent variability in RNA transcription raises mismatches, incomplete hybridization, or disruptions in the reaction in the oligonucleotide panels designed for special TR. This challenge is further compounded when employing multiplexed oligonucleotide panels, as multiple targets increase the likelihood of incorrect binding or incomplete hybridization. In your strategy, we have observed two highly similar motif, such as RNA transcription containing both CUG and CCUG at the same time, may cause cross-hybridization in the oligonucleotide panel only designed for CUG or CCUG. To assess hybridization specificity, a mixture of CUG and CCUG RNAs can be applied to an oligonucleotide panel containing immobilized CAG or CAGG single-stranded DNA probes. The ratio of specific to non-specific hybridization can then be determined. If each TR has to be run separately, it is very inefficient and impractical for broader application.

Version 2:

Reviewer comments:

Reviewer #1

(Remarks to the Author)
- Supporting figure capture rate is captioned as capture rate (1 / hr nM). This requires further explanation, how does one translate that back to molecules per hour?

- Accordinig to Bell et al.'s paper that author mentioned, the maximum sampling rate is 20 kHz if all 16 channels are being used, as stated in the said paper. The author's argument is only valid if each channel of the single amplifier system (e.g. the 16 channel one) operates at 20 kHz. Say for a 0.3 ms translocation signal that the RNA:DNA structure generates, sampling at 100 kHz (which is the lower end and minimum accepted sampling rate in solid-state nanopore system most lab will have to use), you will have 30 data points, if you sample at 20 kHz, you have 6 data points.
What I tried to ask is that if your entire trace has 6 data points, can you still resolve the e,g, 1001 + R spike as you demonstrated throughout (simply put, it all these signals that the paper presented have 6 points, would you still be able to differentiate 1001+R spike signal?
Next, the signal attenuation brought by the usage of low-pass filter as shown cleanly in this paper: https://pubs.acs.org/doi/10.1021/ac901877z, the spike signal at such low sampling frequency will be further attenuated to become almost like a baseline. These are realistic conditions that I do not think the author still able to address, and this technology still require custom solutions on high-end electronics, if the author wants to do real time diagnostic in places like hospital with this technology.

Reviewer #2

(Remarks to the Author)
The authors have addressed all my comments. Thank you!

Reviewer #3

(Remarks to the Author)
The author has adequately addressed my comments. The results are clearly presented and well-supported by experimental data and prior research. Overall, this work is suitable for publication.

Open Access This Peer Review File is licensed under a Creative Commons Attribution 4.0 International License, which permits use, sharing, adaptation, distribution and reproduction in any medium or format, as long as you give appropriate credit to the original author(s) and the source, provide a link to the Creative Commons license, and indicate if changes were made.
In cases where reviewers are anonymous, credit should be given to 'Anonymous Referee' and the source.
The images or other third party material in this Peer Review File are included in the article's Creative Commons license, unless indicated otherwise in a credit line to the material. If material is not included in the article's Creative Commons license and your intended use is not permitted by statutory regulation or exceeds the permitted use, you will need to obtain permission directly from the copyright holder.
To view a copy of this license, visit https://creativecommons.org/licenses/by/4.0/

Response letter to the reviewers for the manuscript:

Quantification of disease-associated RNA tandem repeats by nanopore sensing

Patiño-Guillén, G.[1], Pešović, J.[2], Panić, M.[3], Earle, M.[1], Ninković, A.[2], Petrușca, S.[1], Savić-Pavićević, D.[2,*], Keyser, U.F.[1,*], Bošković, F.[1,*]

[1]Cavendish Laboratory, University of Cambridge, Cambridge, UK

[2]University of Belgrade – Faculty of Biology, Centre for Human Molecular Genetics, Belgrade, Serbia

[3]Institute of Virology, Vaccines and Sera "Torlak", Belgrade, Serbia

*Corresponding authors: fnb24@cam.ac.uk, ufk20@cam.ac.uk, duska@bio.bg.ac.rs

We are grateful to the reviewers for the time devoted to assessing our manuscript. In addition, we appreciate the reviewers' insightful comments and corrections Below, we provide detailed responses to each point raised. We have adapted the manuscript according to the reviewers' suggestions. The reviewers' points are in italic, and our responses are in regular font. The modifications implemented in the manuscript are highlighted in red.

Reviewer #1

- *The study utilised an extensively tried and tested and characterised solid-state nanopore methodology from the research group for repeated expansion detection. From this perspective, the novelty of this paper must stem from design of strategy and how authors adopt this strategy for the proposed detection of repeat expansion. Based on this I have the following comments:*

Thank you for taking the time to review our manuscript and for your comments. We appreciate that you value the novelty in the design and implementation of RNA:DNA for detecting tandem repeat expansions.

Below you can find our replies and changes done to the manuscript that address your comments.

- *The system from publisher listed Boskovic, F. as first author, but in the pdf Patino-Guilllen, G. is the first author?*

Thank you for pointing this out, Patiño-Guillén, G. is the first author of the manuscript and Bošković, F. is the last corresponding author. We have revised the information in the Publisher system to make sure that the authors are listed correctly.

- *Authors referring the hybrid construct as RNA nanostructures but it is in fact a RNA:DNA hybrid nanostructures as the formation of said RNA nanostructures contains more DNA than RNA, since the DNA oligos are longer with an end sticking out for the biotin, this*

confused me as I thought you assembled the entire structures as a RNA:RNA duplex (dsRNA) structure.

To avoid confusions and improve the clarity of the manuscript, we have replaced the term "RNA nanostructures" with "RNA:DNA nanostructures".

- *Please provide a gel of the final RNA:DNA hybrid structures.*

We have produced a new supplementary figure (Figure S36) which shows 1% agarose gel electrophoresis characterization of the final RNA:DNA nanostructures.

Figure S36.

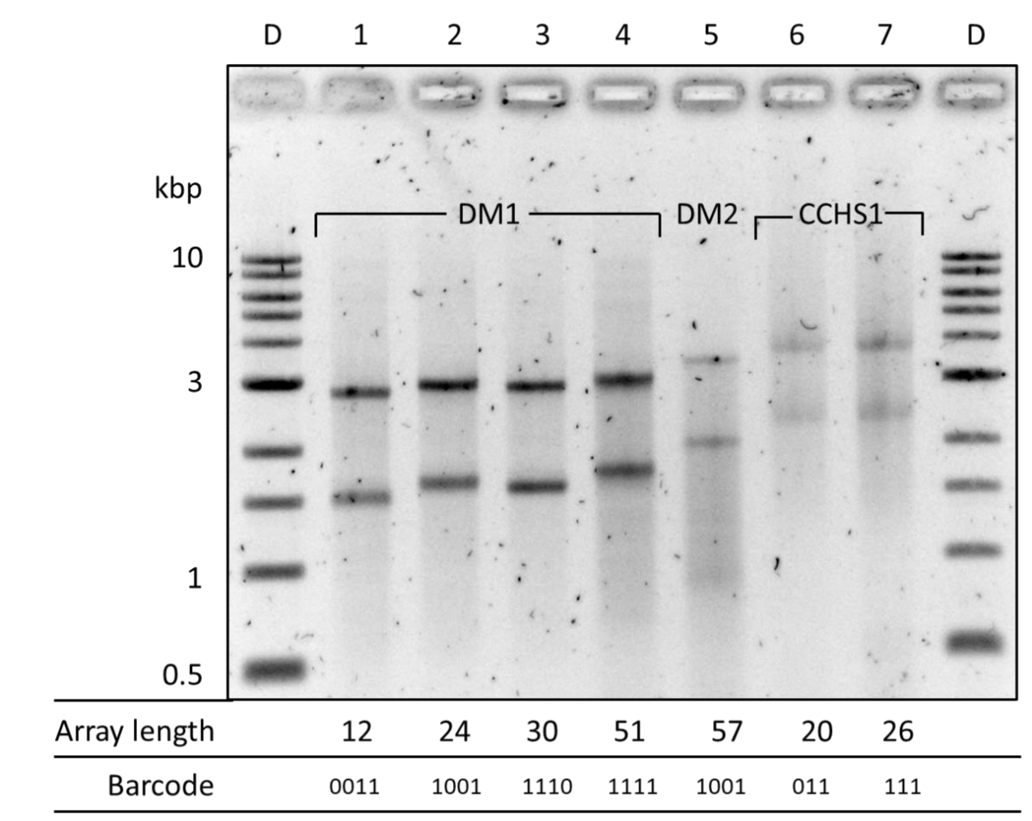

Figure S36. Electrophoretic mobility assay (1% agarose gel) of RNA:DNA nanostructures implemented for characterization of tandem repeats. Lanes 'D' correspond to DNA ladders, which are used as reference, ranging from 0.5 to 10 kbp. Lanes 1 to 4 show nanostructures from transcripts holding 12, 24, 30 and 51 CUG repeats, respectively. Each nanostructure holds a four-digit barcode, where '1' corresponds to 12 consecutive DNA dumbbells and '0' is assigned to RNA:DNA heteroduplex. Lane 5 is an RNA:DNA nanostructure from a transcript with 57 CCUG repeats. Lanes 6 and 7 show nanostructures for 20 and 26 GCN repeats, respectively. Each nanostructure holds a three-digit barcode, where '1' corresponds to 12 consecutive DNA dumbbells and '0' is assigned to RNA:DNA heteroduplex. For each nanostructure, two bands are depicted. The upper band corresponds to the full-length RNA:DNA nanostructure and the lower band originates from the nanostructure of prematurely terminated transcripts.

- *The author assumes that for a say 51 CUG (153 nt) repeats, there will be 5 x $(CAG)_{10}$ (5 x 30 nt) attached to the nanostructure. This is assuming every DNA oligos are at the right place and desired position. Is the authors saying this is an absolute (either full 5 attached, or no attached) or is that more likely to be a distribution of possibilities? This changes how readers interpret the results. So far from the writing, a strong sense of absolution can be sensed. My humble opinion is that the system should fluctuate at least from 4 x $(CAG)_{10}$ to 6 x $(CAG)_{10}$.*

We agree with the reviewer that it is possible to have four or six $(CAG)_{10}$ oligonucleotides hybridized to a 51 CUG repeat array (or equivalent scenarios with other repeat array lengths). When five $(CAG)_{10}$ are hybridized, RNA:DNA base pairing is maximized, giving the lowest free energy and highest stability to the system, making this the most favourable hybridization conformation, as we have shown via Agarose Gel Electrophoresis (Figure S8, now Figure S4, lanes 4 and 6). We clarified in the main text that hybridization of 5 $(CAG)_{10}$ oligonucleotides to a 51 CUG repeat array (or equivalent oligonucleotide hybridization for different repeat array lengths) is not absolute but the lowest free energy state.

(page 3, line 112) "While it is possible for different numbers of $(CAG)_{10}$ oligonucleotides to hybridize to the repeat array, the arrangement where the number of oligonucleotides maximizes base pairing provides the lowest free energy and greatest stability[42], as demonstrated *via* agarose gel electrophoresis (**Figure S4**). Therefore, the binding of five $(CAG)_{10}$ oligonucleotides to 51 CUG repeats correspond to the most favourable assembly conformation."

- *Constructions of the nanostructures, Figure S8 tested some ranges: 3 times excess, and then 9 times excess to find the range to fully cover the CUG expansion region, then the construction of the nanostructures utilise 5 times excess. Why the difference?*

  Additionally, do you mean 5 times excess on all oligos in all regions? In a sequence specific region like the region outside the repeat of 1 RNA molecule, you have a roughly 1 region (say 10 nt) to 5 oligos of 10 nt. For the expansion region of 1 RNA molecule, a 5 repeat region will take 5 oligos of 10 nt, in a folding excess of 5 fold excessive DNA oligos, the expansion region is not in excess. Which from Figure S8, you produced multiple isoforms.

In Figure S8 (now Figure S4), a nine-fold excess corresponds to 3:1 ratio between $(CAG)_{10}$ and the available binding sites for full hybridization of the same array. A three-times excess corresponds to a 1:1 ratio in terms of $(CAG)_{10}$ and the available binding sites for full-length coverage of a $(CTG)_{30}$ repeat array.

During nanostructure assembly, we ensure that every available binding site along the entire transcript is supplied with oligonucleotides at a five-fold excess relative to the potential binding sites, in order to guarantee efficient hybridization. We could also have used lower excess of just three-times as reported previously (Bošković, F., Nat Chem, 2022

and Patiño-Guillén, G., Nat Commun, 2024). We have expanded our Methods section and the Supplementary Figure S8 (now Figure S4) for clarity.

(Figure S4) "Lane 3: $(CTG)_{30}$ + $(CAG)_{10}$-biotin in ~ 1:3 ratio hybridized in 100 mM LiCl. The 1:3 molar excess corresponds to a 1:1 excess of $(CAG)1_{0}$-biotin with respect to the available binding sites for full hybridization in the 30 CTG array. Lane 4: $(CTG)_{30}$ + $(CAG)_{10}$-biotin in ~ 1:9 ratio hybridized in 100 mM LiCl. The 1:9 molar excess corresponds to a 1:3 excess of $(CAG)_{10}$-biotin with respect to the available binding sites for full hybridization in the 30 CTG array."

(Page 16, line 454) "For the tandem repeat labelling oligos, a five-fold excess relative to the available binding sites in the repeat array ensures complete RNA–DNA hybridization. A reduced oligonucleotide excess of threefold can also be used, while still yielding efficient hybridization and nanostructure assembly[30]."

- *I can see some reversible clogging, the events are sparse, can the authors indicate how long the assay on average will be run? 1 hour, 2 hours, 3 hours, overnight? To collect enough data for this assay, say 100 events including false positives.*

At our experimental conditions, the number of translocations depends on the concentration of the nanostructures. Experiment duration can be adjusted according to the concentration. We have included representative measurement durations for each nanostructure in this work in new Table S20.

Table S20.

Representative measurement durations and counts of linear RNA:DNA nanostructures detected.

Figure	Sample	Experiment duration (h)	Unfolded events	Number of nanopores	Measurement termination
**1**	51 CUG and 57 CCUG RNA:DNA nanostructure	~2	~100	1	Measurement was stopped
**2**	12, 24, 30, 51 CUG RNA:DNA nanostructure mix	~6	~400	1	Measurement was stopped
**3**	20 and 26 GCN RNA:DNA	~4	~120	1	Measurement was stopped

	nanostructure mix				
**4**	mRNA and 34 CUG mRNA RNA:DNA nanostructure	~1 to 8	~10	3	Nanopore clogging
**S32**	MS2 nanostructure	~1	~20	1	Nanopore clogging

- *Figure 1a, maybe clearly label which end is the 5′.*

We have modified Figure 1a and added the reviewer's suggestion.

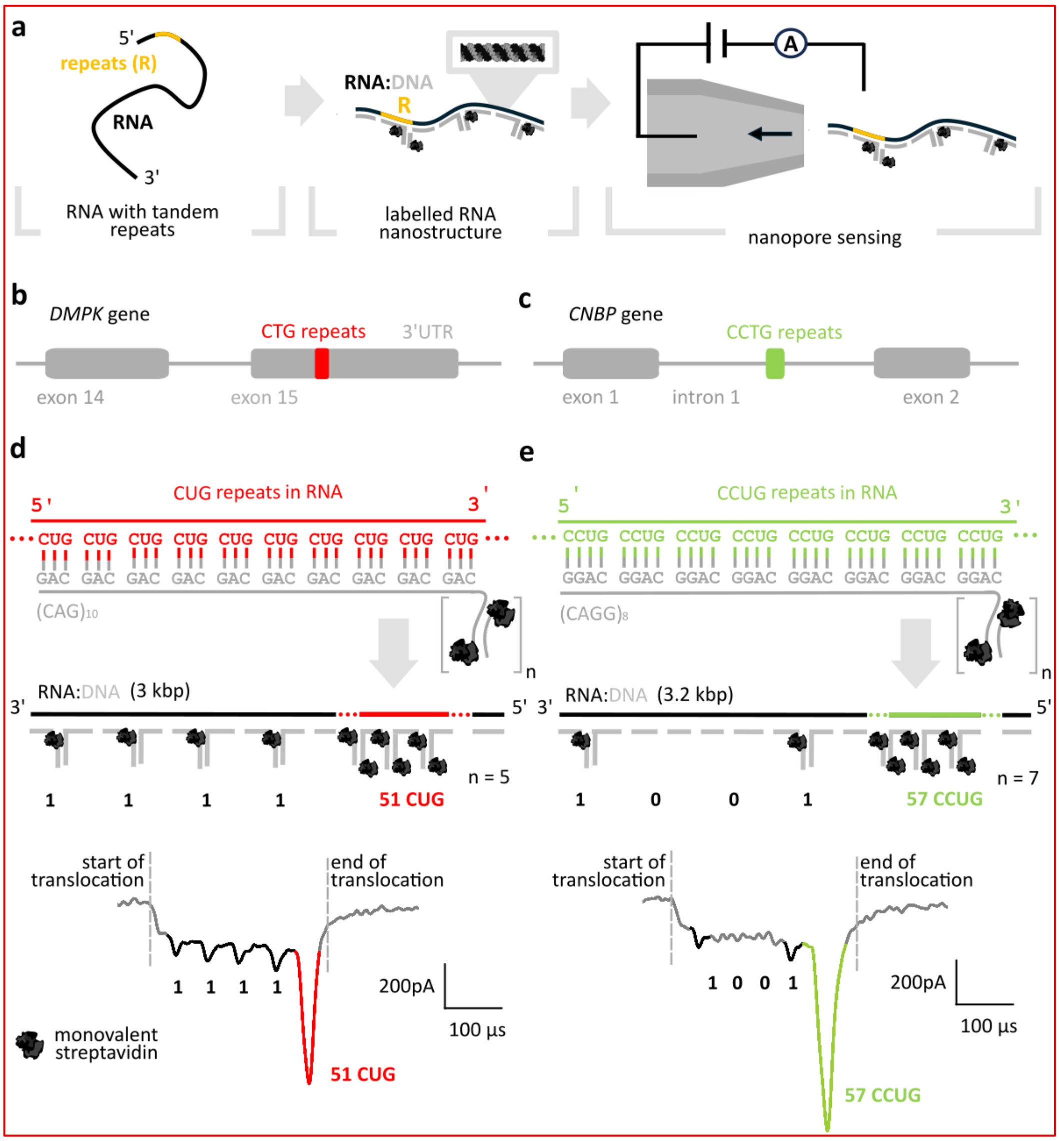

- *Figure 2, placement of letter d is confusing as it merges into panel 2b, the box label from 2b can be moved to a better position to avoid confusion.*

We have modified Figure 2d and added the reviewer's suggestion.

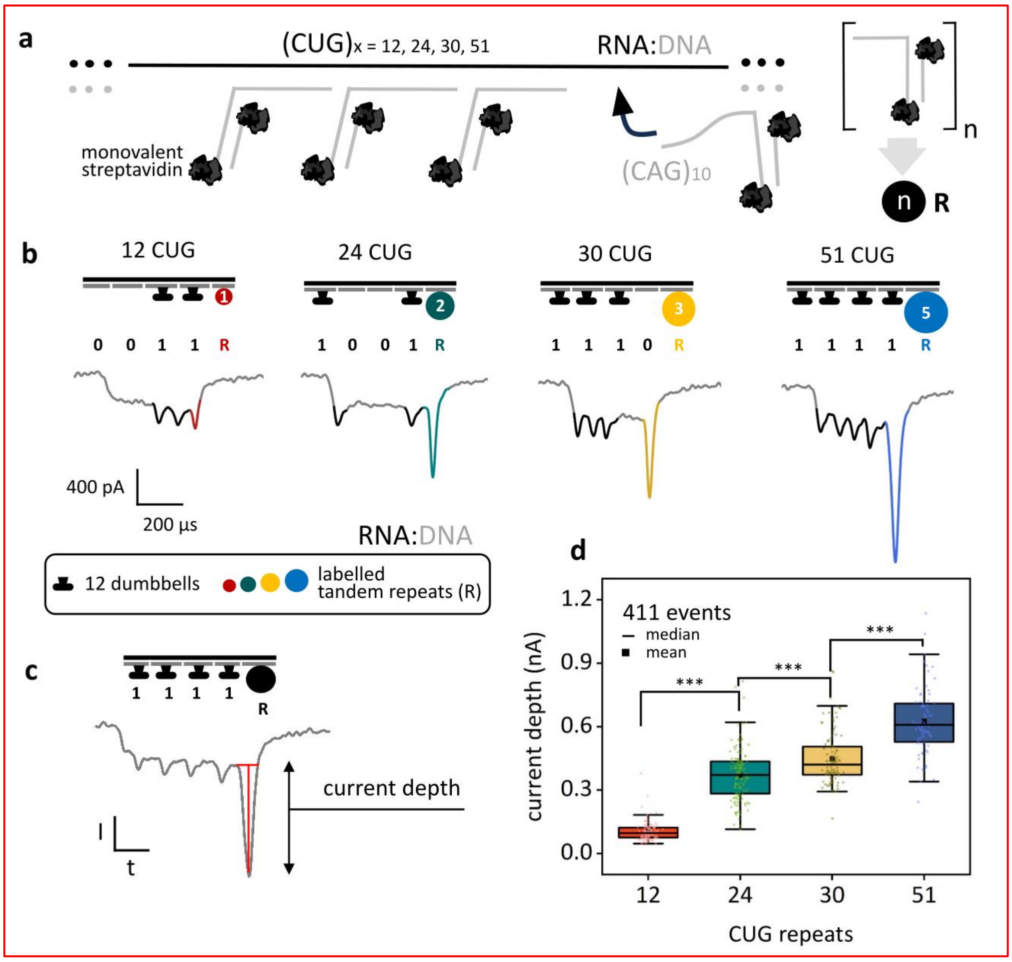

- *2d utilises Welch's t-test, for multiple data comparison, an ANOVA should be used in multiple comparison to minimise Type-1 error.*

We have included ANOVA statistical analysis for the data in Figure 2d and Figure S17 and we have added a supplementary table, Table S22, with the Tukey's Honest Significant Difference test between populations. We implemented the following modifications:

(page 8, line 212) "A one-way ANOVA test f-ratio value is 213.9 and the p-value is < 0.00001."

(Figure S17) "A one-way ANOVA test f-ratio value is 163.3 and the p-value is < 0.00001."

(page 18, line 520) "The Tukey's Honest Significant Difference test (HSD) for distributions in **Figure 2** and **Figure S17** are included in **Table S22**."

Table S22

Tukey's Honest Significant Difference test (HSD). Distributions are labelled as T_n, where n correspond to the repeat array length. Q represents the critical value obtained from the Studentized Range Distribution table and p the p-value.

HSD for data in Figure 2d (spike depth) and data in Figure S17 (spike area)

Figure 2d (spike depth)		
**Pairwise Comparisons**	**$HSD_{0.05}$ = 0.0435**<br>**$HSD_{0.01}$ = 0.0529**	**$Q_{0.05}$ = 3.6482**<br>**$Q_{0.01}$ = 4.4303**
**T_{12}:T_{24}**	0.27	Q = 22.34 (p < 0.00001)
**T_{12}:T_{30}**	0.34	Q = 28.83 (p < 0.00001)
**T_{12}:T_{51}**	0.52	Q = 43.59 (p < 0.00001)
**T_{24}:T_{30}**	0.08	Q = 6.49 (p = 0.00003)
**T_{24}:T_{51}**	0.25	Q = 21.25 (p < 0.00001)
**T_{30}:T_{51}**	0.18	Q = 14.76 (p < 0.00001)
Figure S17 (spike area)		
**Pairwise Comparisons**	**$HSD_{0.05}$ = 1.0964**<br>**$HSD_{0.01}$ = 1.3314**	**$Q_{0.05}$ = 3.6482**<br>**$Q_{0.01}$ = 4.4303**
**T_{12}:T_{24}**	3.90	Q = 12.98 (p < 0.00001)

**T_{12}:T_{30}**	5.94	Q = 19.78 (p < 0.00001)
**T_{12}:T_{51}**	9.88	Q = 32.89 (p < 0.00001)
**T_{24}:T_{30}**	2.04	Q = 6.80 (p = 0.00001)
**T_{24}:T_{51}**	5.98	Q = 19.91 (p < 0.00001)
**T_{30}:T_{51}**	3.94	Q = 13.11 (p < 0.00001)

- *With regards to transfection and extraction detection, transfection can cause different levels of expression of certain construct, thus the mRNA (GFP in this case) will and should be the dominant species in the RNA pool without need to further refine the RNA samples. I wonder if authors can provide some comments on how this can be translated into real scenario, e.g. the diagnostic of say DM1, i.e. you isolated some levels of total RNA from a patient or animal model, and you perform the nanopore with this assay, the transcript is probably not abundance in your total RNA pool, since the issue is not that the disease caused over expression of this mRNA?*

We have expanded our discussion to address this point. Detection of messenger RNAs with comparable expression have been demonstrated in human total RNA before (Bošković, F., Nat Chem, 2022). The acquisition time can be scaled down by simultaneous ionic current measurements in solid-state nanopores, as previously demonstrated (Bell, N.A.W. et al., Lab on a Chip, 2013). We have expanded our manuscript text to address this point.

(Page 11, line 315) "RNA:DNA nanostructure assembly and subsequent characterization *via* nanopores is not limited to the detection of overexpressed or highly abundant RNA. Characterization of mRNA with low copy numbers has been demonstrated previously[30,44]. Alternatively, the nanostructure capture rate and throughput can be increased through simultaneous ionic current measurements using multiple nanopores[54]."

- *The SI is quite a big document, I suggest having all the sequence as a separate pdf file or excel file (if allowed).*

We appreciate the reviewer's suggestion, but we prefer to keep everything in one supplementary document.

- *Data availability said is provided, but not clear on how are these being provided? Repository?*

The source data have now been provided to the journal as a supplementary dataset in the current submission, and the depository link will be provided with the article as requested by the journal.

- *Overall, while unique in trying to tackle the problem of repeat expansion detection, there are some caveats remain to be addressed or acknowledged the limits.*

We thank again the reviewer for highlighting uniqueness of our approach and for the insightful comments that have helped us to improve the quality of manuscript.

Reviewer #2

- *I'm excited to read a novel method for detecting short tandem repeat expansions. As noted but the authors, while long-read sequencing has been incredibly valuable in this field, the DNA input requirements and expense have hindered adoption into clinical labs. This method may provide the basis for a new approach to testing these loci.*

We appreciate and share the reviewer's excitement towards our characterization method for tandem repeat expansions. We provide the point-by-point response below.

Major:

- *Why did you choose DM1, DM2, and CCHS1? How well do you expect this technique to generalize to additional STR loci? For example, do you expect it to perform well on other motifs, especially those with more extreme GC content, e.g. FAME loci (20% GC) or C9orf72 (100% GC)? Would you expect it to work on VNTRs (7+ bp motifs)?*

DM1, DM2, and CCHS1 were chosen as clinically relevant loci with different repeat contexts to validate the method. In addition, these tandem repeat expansions are available from our plasmid collection, and our protocols were optimized for their cloning.

The technique can generalize to additional STRs (CUG and CCUG repeats are already 67% and 75% GC content, respectively), but loci with 100% GC content (e.g., *C9orf72*) or low GC content (e.g., FAME) may require further optimization to enhance the hybridization efficiency. For VNTRs with longer motifs, the approach remains feasible. We have highlighted these points in the Discussion section:

(Page 13, line 344) "We characterized CUG and CCUG repeats as reliable validation models for quantification of tandem repeat arrays with variable motif lengths. We studied GCN repeats for validation of our model for non-uniform arrays and to demonstrate its resolution holds clinical relevance. Our approach may also be applicable to other STRs with 100% (e.g. C9orf72) and low GC content (e.g. FAME associated loci), and to variable number tandem repeats, provided that further optimization of the hybridization of oligonucleotides is performed."

- *"The tandem repeat size distributions agree with the spread observed for the repeat sizes of the initial DNA plasmids used for RNA synthesis." Could you please plot the distribution of (inferred) allele sizes observed for each experiment and visually compare to the expected size? The data presented is almost exclusively in "signal" space. This would be critical to assess the degree of stutter/mosaicism as well as estimating accuracy.*

Indeed, the repeat array size can be inferred and plotted from the ionic current readout. We have included an additional Supplementary Figure, Figure S15, where we correlate the repeat number with the ionic current observables.

(Page 8, line 218) "In **Figure S15**, we have included visual representation of the allele sizes from the ionic current readout…".

Figure S15

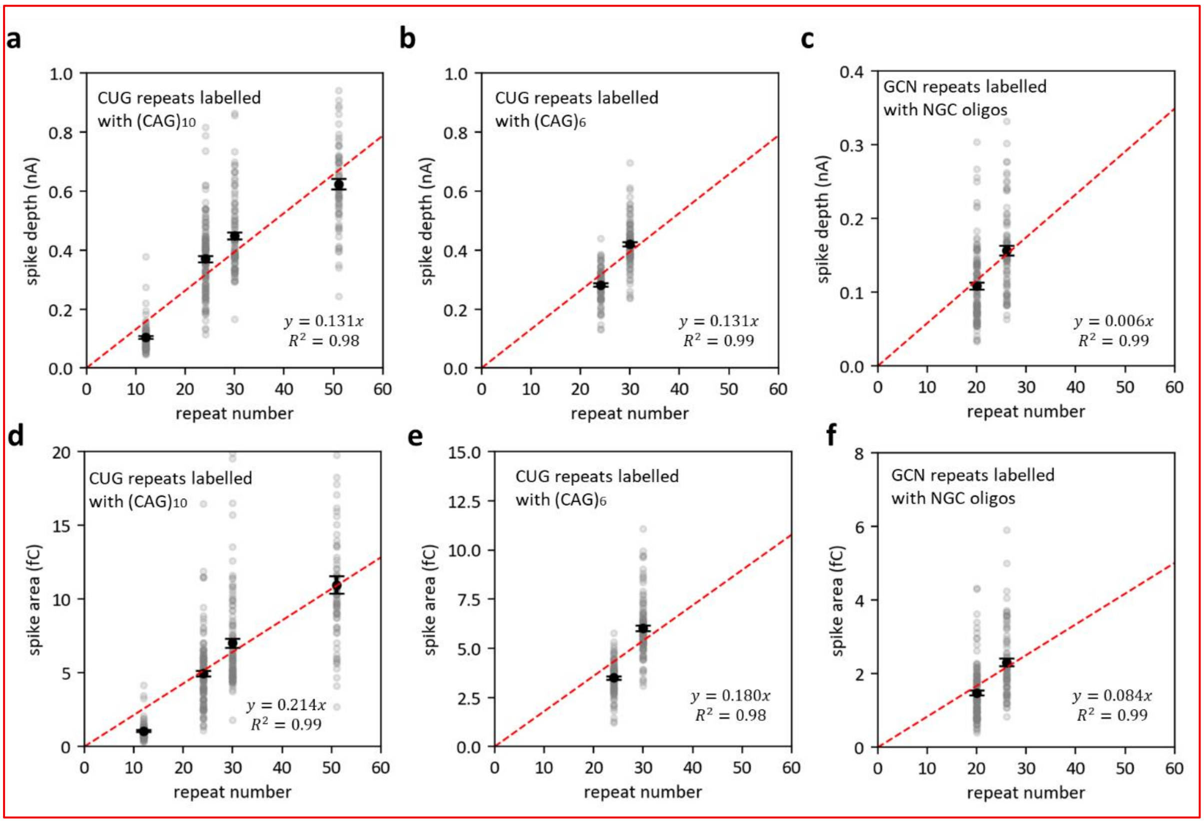

Figure S15. Correlation of repeat array length to current spike observables. The expected repeat array length based on the spike depth for characterization of **a** 12, 24, 30 and 51 CUG repeats with $(CAG)_{10}$ oligonucleotides. **b** 24 and 30 CUG repeat arrays with $(CAG)_6$ oligonucleotides. **c** 20 and 26 GCN repeats with NGC oligonucleotides. The expected repeat array length based on the spike area for characterization of the same CUG repeats with **d** $(CAG)_{10}$ and **e** $(CAG)_6$ oligonucleotides, and **f** the same GCN repeats with NGC oligonucleotides. The mean of each distribution is indicated. Error bars indicate standard error.

- *Does this technique allow you to directly estimate the allele size for each sample, or does it focus on distinguishing between size categories (pathogenic vs benign)? It appears that only one intermediate or pathogenic-sized allele was tested for each locus. If relevant, could you please elaborate on what would be needed to estimate allele sizes, including those much larger than those tested. How would this method handle interruptions and motif variations?*

The nanopore observables allow direct estimation of the allele size in each sample. The number of repeats directly correlates with the number of streptavidin proteins bound to the nanostructure, and therefore with the spike depth or area in the ionic current readout (Bošković, F., Nat Chem, 2022).

We have included a new Supplementary Figure S16, which shows repeat arrays centred on 12 CUG repeats, a broader repeat array with 51 CUG repeats, and a heterogenous CUG repeat arrays containing up to 89 repeats to exemplify the ability of the method to detect size heterogeneity typically seen in patient cells due to repeat instability.

(Page 8, line 219) "We also show the characterization of a repeat array with significant size heterogeneity typically seen in patient cells due to repeat instability in **Figure S16**."

Figure S16

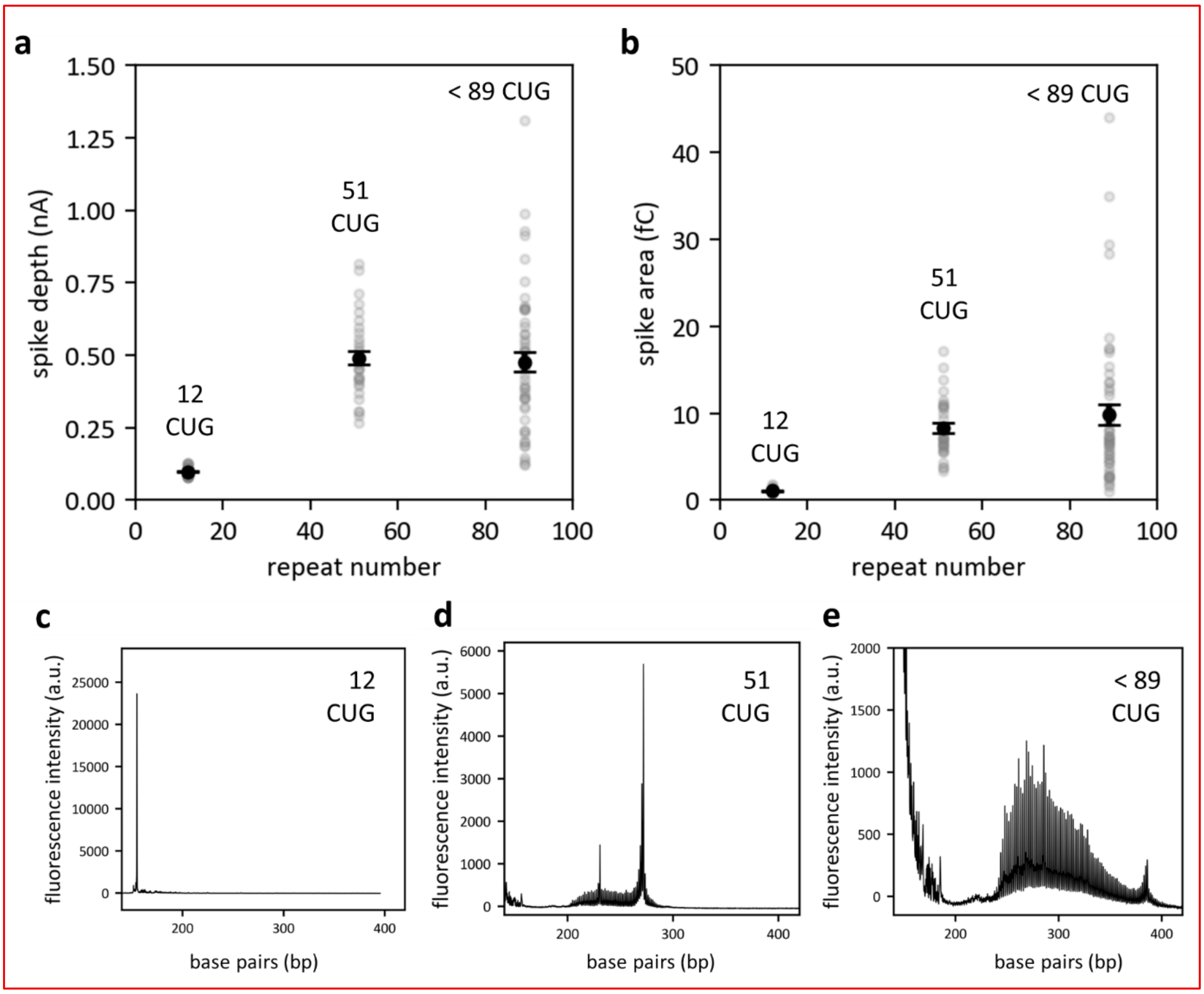

Figure S16. Correlation of repeat array length to current spike observables including the spike depth and spike area. We show the spike **a** depth and **b** area for a CUG repeat array of 12 repeats, 51 repeats and a mix of repeat lengths up to 89 repeats. The mean of each distribution is indicated. Error bars indicate standard error. Panels **c**, **d**, and **e** show the fragment analysis for each repeat array, which were performed using GeneScan 600 LIZ as the size standard.

At present, this work does not address repeat array interruptions. We are currently preparing follow-up work focused on mapping interruptions in tandem repeat arrays, which will then be suitable for VNTRs characterized by great motif diversity.

- *While code was provided (https://github.com/maxearle/nas2), no usage documentation was available. Sample input data would also be needed to test it.*

We have updated the GitHub attachment with '*read me*' documentation that facilitates smooth implementation of the resources and we have included sample input data.

Minor:

- *Line 34: This is a little out of date. I would put it at 65-68 STR loci are associated with mongenic conditions, depending on how you count. So it would be more accurate to say more than 60 and cite a more recent paper. Also, by my count, ~30 STR of those loci are coding. If I'm understanding correctly, this technique would only work on coding STRs, so this is the true number of loci that are in scope. You may consider adding the total number of coding STRs (not just pathogenic ones) to demonstrate the potential scope of your work. Or, could the method be adapted to DNA?*

Our method applies to transcribed STRs, not only coding STRs. For example, in our work we show characterization of CUG and CCUG repeats which are transcribed but are located in non-coding regions of *DMPK* and *CNBP* genes, respectively. We also characterize GCN repeats which are in a coding region of *PHOX2B* gene. We have updated references and revised the introduction to reflect the reviewer's input on the current number of repeat expansion disorders ("over 60 monogenic disorders"). As clarified in the text, the method presented here is specifically focused on STRs within transcribed regions of genes. The following changes have been introduced:

(Page 1, Line 34) "Expansions of short tandem repeats are implicated in over 60 monogenic disorders, of which roughly half occur within protein-coding regions of genes, and are collectively known as repeat expansion disorders (REDs)[2-5]. REDs affect 1 in 3,000 individuals[2,3]."

(Page 2, Line 74) The method is suitable for characterization of transcribed small tandem repeats.

- *Line 40-41: "The severity and onset of REDs [typically] depend on the number of repeats". For some diseases, it affects onset, in some severity, and in others, both. Interruptions and motif play a major role.*

We have added the reviewer's suggestion:

(Page 1, line 41) "The severity and onset of REDs typically depend on the number of repeats and interruptions…"

- *Line 54: You should include PacBio sequencing (both WGS and PureTarget) in this background.*

We have now included background on characterization of tandem repeats *via* PacBio sequencing.

(Page 1, line 60) “Similarly, PacBio sequencing has been used to characterize DNA tandem repeats at whole genome scale and targeting specific loci *via* PacBio *PureTarget*[24,25]. PacBio offers long reads and high consensus accuracy though HiFi reads[24]. However, it also demands for high DNA inputs[26], and high error rates prevail for repetitive or GC-rich motifs which demand specialized algorithms for data processing[27,28].”

- *Line 70-71: “trinucleotide repeats” DM2 is a 4bp motif*

We changed the text and removed “trinucleotide repeats”.

(Page 2, line 75) “We characterize RNA tandem repeats arrays of varying sizes derived from the loci associated with DM1, DM2, and CCHS1, showing a resolution of 18 nucleotides.”

- *Line 349: “our method paves the way for detecting interruptions”. Could you please explain this point with regard to the experiments presented in this paper? How would the method be adapted to do this?*

We thank the reviewer for this point. At present, this work does not address repeat array interruptions. We are currently preparing follow-up work focused on mapping interruptions in tandem repeat arrays. We have removed the statement pointed out by the reviewer.

Reviewer #3

(Remarks to the Author):

- *This study uses present a single-molecule nanopore-based strategy that enables direct quantification of tandem repeats in native RNA,RNA nanostructures enabled detect and distinguish disease-associated repeats in DM1, DM2, and CCHS1.*

We thank the reviewer for acknowledging the capabilities of our method.

- *This study has multiple major limitations:*
  In your paper, you describe the use of DNA oligonucleotides and tandem repeat–containing RNA to construct RNA nanostructures for copy number assessment. Your approach involves in vitro transcription from DNA followed by hybridization with complementary DNA oligonucleotides. Given the high diversity and complexity of tandem repeat sequences, you present only a few examples to illustrate the feasibility of the method, and the DNA oligonucleotides used in these experiments may not be broadly applicable. Could you elaborate on how you envision addressing the sequence diversity and throughput challenges in practical applications?

We agree that the high diversity and complexity of tandem repeat sequences pose challenges for broad applicability. Our current work demonstrates feasibility on selected disease-relevant loci, but we envision extending the method in two complementary ways. First, oligonucleotide design can be tailored to specific repeat motifs, with optimization of hybridization conditions to accommodate sequence composition. Second, throughput can be increased by implementing multiplexed oligonucleotide panels and parallelized nanopore measurements, as demonstrated previously (Bell, N.A.W. *et al.*, Lab on a Chip, 2013). These strategies should enable adaptation of the approach to a wide range of repeat motifs and support practical applications at scale. We have added text in the Discussion to highlight these future directions.

(Page 13, line 344) ""We characterized CUG and CCUG repeats as reliable validation models for quantification of tandem repeat arrays with variable motif lengths. We studied GCN repeats for validation of our model for non-uniform arrays and to demonstrate its resolution holds clinical relevance. Our approach may also be applicable to other STRs with 100% (e.g. C9orf72) and low GC content (e.g. FAME associated loci), and to variable number tandem repeats, provided that further optimization of the hybridization of oligonucleotides is performed."

(Page 11, line 315) "RNA:DNA nanostructure assembly and subsequent characterization *via* nanopores is not limited to the detection of overexpressed or highly abundant RNA. Characterization of mRNA with low copy numbers has been demonstrated previously[30,44]. Alternatively, the nanostructure capture rate and throughput can be increased through simultaneous ionic current measurements using multiple nanopores [54]."

- *In Figure 2d and Figure 3b, you demonstrate that different copy numbers of the motif repeats, identified using RNA nanostructures, are associated with varying degrees of ionic current reduction. However, it is worth noting that even among RNAs with the same repeat copy number, the current depth exhibits considerable variability, raising doubts about its ability to precisely quantify changes in tandem repeat copy numbers.*

In this work, the spike depth variability mainly originates from the heterogeneity and dynamic nature of the repeat arrays (Hannan, A. J., Nat Rev Genet, 2018). There is also intrinsic spike depth variance associated to streptavidin sensing. The overall spike depth variability observed in the characterization of the repeat arrays correlates with the variability of fragment analysis, presented in Figure S10 and Figure S21. To expand on this, we have included a new Supplementary Figure, Figure S16, which shows characterization of repeat arrays centred on 12 CUG repeats, a broader repeat array with 51 CUG repeats, and a mix of CUG repeat arrays with sizes up to 89 repeats. The nanopore variability agrees with independent fragment analysis as expected.

(Page 8, line 219) "We also show the characterization of a repeat array with significant size heterogeneity typically seen in patient cells due to repeat instability in **Figure S16**."

Figure S16

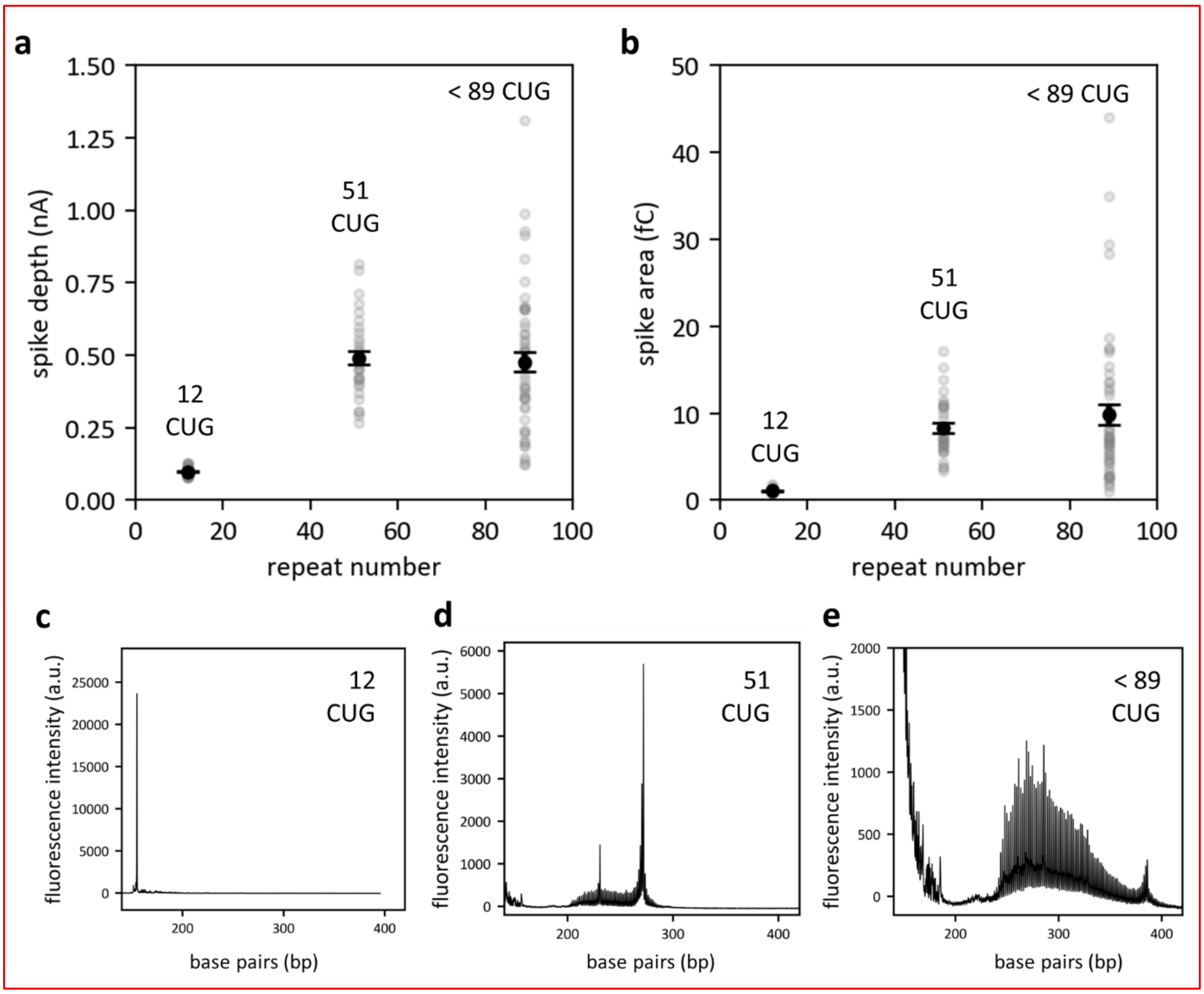

Figure S16. Correlation of repeat array length to current spike observables including the spike depth and spike area. We show the spike **a** depth and **b** area for a CUG repeat array of 12 repeats, 51 repeats and a mix of repeat lengths up to 89 repeats. The mean of each distribution is indicated. Error bars indicate standard error. Panels **c**, **d**, and **e** show the fragment analysis for each repeat array, which were performed using GeneScan 600 LIZ as the size standard.

- *First, on page 2, line 94, you define abnormal expansion of the CNBP gene in DM2 as greater than 57 CCTG repeats. However, according to the most recent literature, the pathogenic threshold is generally considered to exceed 75 repeats. Could you clarify whether selecting 57 repeats as the cutoff for abnormal expansion is appropriate?*

Thank you for pointing this out, because there is no consensus about the clinical relevance of alleles in the range of 55 to 75 repeats. The reviewer is correct that the majority of literature, including the latest papers, defines the pathogenic cutoff for DM2 as greater than 75 CCTG repeats, relying on the paper reporting on the discovery of DM2 mutation (Liquori *et al.*, Science, 2001), although a few DM2 patients with expansions in the range of 55 to 75 repeats have been identified (Baschinski *et al.*, Neurology, 2009). Our reference (Meola, Acta Myol, 2020) combines above mentioned findings. The other paper we have cited (Schoser, GeneReviews, 2020) considers the range of ~55-74 CCTG repeats as premutation or pathogenic with uncertainty, due to incomplete penetrance (Wendlandt *et al.*, Neurol Genet, 2024). In addition, there is no consensus in databases for repeat expansions. For example, gnomAD and STRipy define CNBP alleles ≥55 repeats as pathogenic while STRchive defines alleles ≥75 as pathogenic. In this manuscript, we also rely on our experience in DM2 genetic testing and a close collaboration with experienced neurologists, since Serbia is a country with the second-highest rate of DM2 (Peric *et al.*, J Neuromuscul Dis, 2024). In our paper (Ivanovic *et al.*, Neurol Sci, 2023), we considered individuals as DM2 positive with more than 50 repeats based on long-term follow-up and development of typical DM2 clinical presentation. Therefore, the lower DM2 pathological range is still a matter of debate.

In our manuscript, we referred to our repeat size as “expansion” because this range includes both premutation (26–75 repeats) and the smallest pathogenic alleles reported as 55-75 repeats. Our intention was to capture the full spectrum of expanded (non-normal) alleles, rather than to suggest that >55 repeats alone represents the hard cutoff for pathogenic expansions. The recent 2020 DM2 update and our experience referred to well-diagnosed DM2 patients with 55-75 CCTG repeats. To avoid confusion, we can revise the text to clarify this distinction: >75 repeats as pathogenic cutoff, and 26–75 as premutation, with “expansion” referring collectively to both categories.

(Page 3, line 100) “For DM2, the wild-type tandem repeat array contains fewer than 26 repeats; alleles with 26 to 75 repeats correspond to a premutation. A repeat length of over

75 defines the pathogenic cutoff. The term "expansion" collectively refers to both the premutation and pathogenic categories (**Figure 1c**)[40,41]."

- *Furthermore, non-pathogenic CCTG expansions are often interrupted by GCTG or TCTG motifs. Your methodology does not appear to describe how such interruptions within the CCTG repeats region were identified or differentiated. In addition, could you elaborate on whether the insertion of GCTG or TCTG motifs affects the ionic current signals measured through nanopores, and how the magnitude of such reductions can be distinguished from those caused by CCTG repeats? Clarifying this would be important for accurately determining the true number of CCTG repeats.*

Currently, our method does not distinguish interruptions. Given our current oligonucleotide design, the presence of a GCTG or TCTG motif in a CCTG repeat array would still enable the hybridization of a $(CAGG)_8$ oligonucleotides with a single mismatch, which would produce negligible variation in the ionic current readout. We are currently preparing follow-up work focused on detecting and mapping interruptions in tandem repeat arrays. We have included a statement addressing the reviewer's comment to provide more context of the current capability of the method.

(Page 4, line 143) "Interruptions or mismatches in CCUG repeat arrays, such as the inclusion of GCTG or TCTG motifs, do not prevent the binding of $(CAGG)_8$ oligonucleotides and are not expected to cause significant variation in the ionic current readout with the present labelling strategy."

- *On page 17, line 497, you state that plasmids containing 10, 18, and 34 CTG repeats were obtained and transfected. However, in the subsequent analyses, current spikes corresponding to the 10 and 18 CTG repeats were not reported. It would be helpful if you could clarify whether these data were collected but not shown, or elaborate on the reasons for their absence. Including these results, if available, would provide a more complete understanding of the relationship between repeat copy number and current spike patterns.*

Here, we have focused on demonstrating the applicability of our sensing approach in a complex mixture of human total RNA. It was an honest mistake to include plasmids with 10 and 18 CTG repeats in our Methods section. We have now corrected the Methods section.

(Page 17, line 529) "In brief, the IIs restriction enzyme sites allowed the creation of a stepwise and seamless elongation of repetitive sequences and we have obtained the plasmid with 34 CTG repeats."

Response letter to the reviewers for the manuscript:

Quantification of disease-associated RNA tandem repeats by nanopore sensing

Patiño-Guillén, G.[1], Pešović, J.[2] , Panić, M.[3], Earle, M.[1], Ninković, A.[2], Petrușca, S.[1], Savić-Pavićević, D.[2,*], Keyser, U.F.[1,*] , Bošković, F.[1,*]

[1]Cavendish Laboratory, University of Cambridge, Cambridge, UK

[2]University of Belgrade – Faculty of Biology, Centre for Human Molecular Genetics, Belgrade, Serbia

[3]Institute of Virology, Vaccines and Sera "Torlak", Belgrade, Serbia

*Corresponding authors: fnb24@cam.ac.uk, ufk20@cam.ac.uk, duska@bio.bg.ac.rs

We are grateful to the reviewers for the time devoted to assessing our manuscript. In addition, we appreciate the reviewers' insightful comments and corrections Below, we provide detailed responses to each point raised. We have adapted the manuscript according to the reviewers' suggestions. The reviewers' points are in italic, and our responses are in regular font. The modifications implemented in the manuscript are highlighted in red.

REVIEWER COMMENTS

Reviewer #1 (Remarks to the Author):

The revised manuscript addressed concerns that I raised from the last round of review. Now the revised edition lead to some more questions for the author to clarify:
We thank the reviewer for their careful reading of the revised manuscript and for the detailed technical comments. We address each point below.

1. The newly added S36 of the gel structure shows two bands with one near 3 kbp and one closer to 1 kbp… do you observe translocation events of both fully formed and truncated RNA:DNA nanostructure? If so what proportion… Same question with the mRNA detection, how often do you detect the RNA:DNA labelled construct? or do you only detect the construct?

As the reviewer points out, assembly protocols retain both the full-length RNA:DNA nanostructures and the nanostructures from prematurely terminated transcripts. We now explicitly state this in the revised manuscript.

(Page 2, line 89): "*In vitro* transcription yields full-length and prematurely terminated transcripts[35]. In this study, we use the full-length RNA to characterize tandem repeats."

The distinction, quantification and characterization of truncated transcripts is extensively described in our previous work Patino-Guillen et al., Nat Commun 2023. Importantly, these species are readily distinguishable in nanopore recordings: our analysis filters events by translocation time, charge deficit, and barcode structure, such that only correctly assembled full-length RNA:DNA nanostructures are used for tandem repeat readout (Patino-Guillen et al., Nat Commun 2023; Boskovic et al., Nat Chem 2022). We added workflow as shown in new Figure S40 and modified the text:

(Page 19, line 537): "In **Figure S40**, we include a detailed workflow illustrating the quantitative distinction of truncated RNA products and the constructs assembled from full-length RNA."

Figure S40.

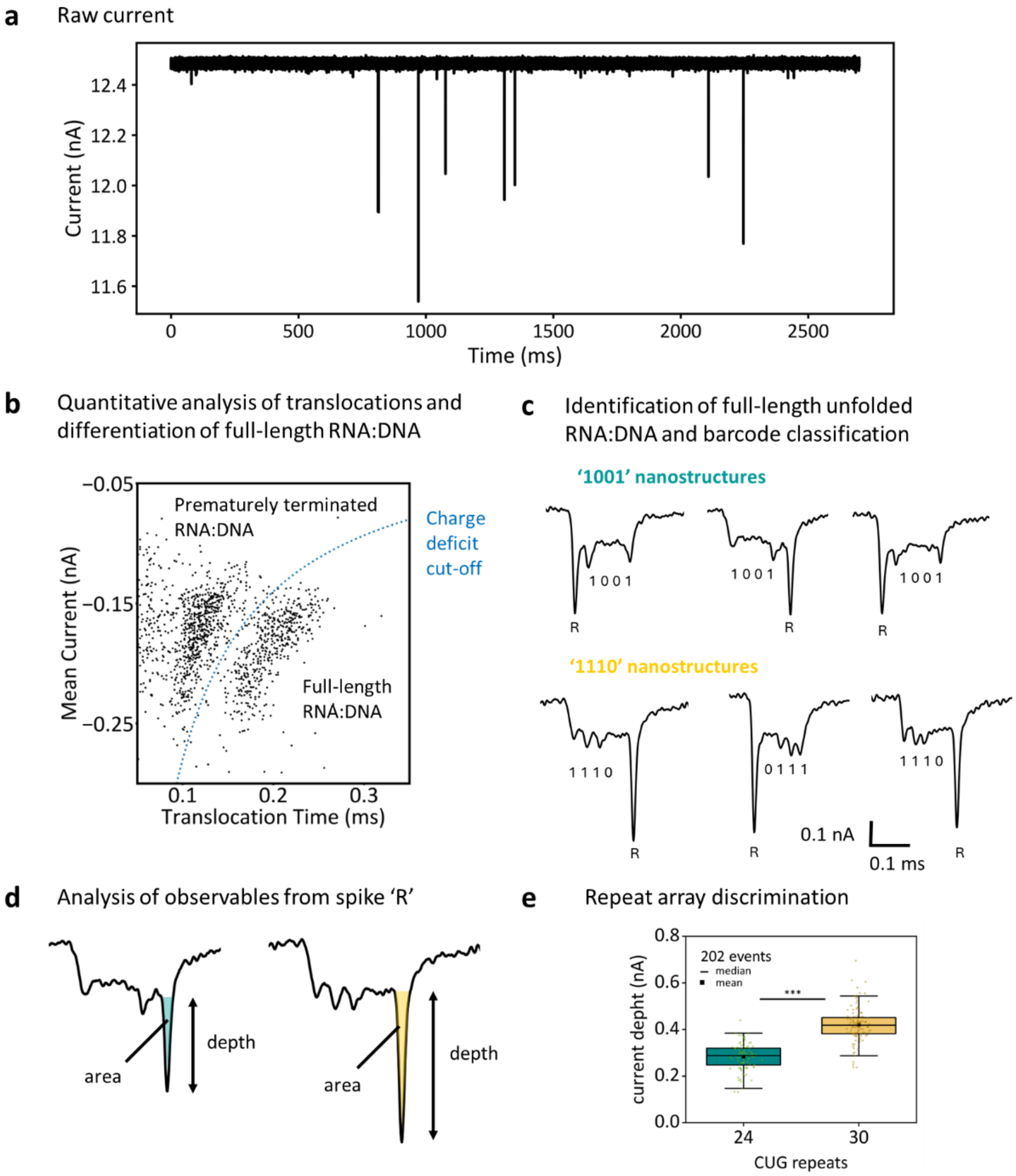

Figure S40. Step-by-step nanopore data analysis of tandem repeat arrays. **a** An example segment of the raw ionic current trace is shown for multiplexed characterization of transcripts containing 24 and 30 tandem repeats. Translocation events are identified using custom LabVIEW software based on simple thresholding. Typical parameters include a translocation duration >0.05 ms, a charge deficit of 10–400 fC, and a mean event current below −100 pA. These criteria enable reliable detection of events corresponding to RNA:DNA nanostructures. **b** Further quantitative analysis of event features (translocation time, charge deficit, mean current) is used to distinguish full-length nanostructures from prematurely terminated or fragmented constructs. **c** Using the nanostructure design and translocation time, unfolded constructs are classified by barcode. In this example, the 24-repeat nanostructure carries a '1001' barcode, whereas the 30-repeat nanostructure carries a '1110' barcode. **d** Finally, the spike depth and area associated with the tandem repeat region are quantified, **e** enabling discrimination of repeat array length.

For mRNA experiments, we detect mRNA RNA:DNA nanostructures and unlabeled single-stranded RNA in nanopore translocations. The latter produces non-specific ionic current drops that are highly distinguishable from the mRNA nanostructure readout, as shown in exemplary current trace of **Figure S33** (Earle, M. et al., Analytical Chemistry 2025, and Chau, C. et al., ACS Nano 2022). In this work we focused on the characterization of the RNA:DNA nanostructures and disregarded the analysis of unlabeled RNA. We have reported the capture rate for mRNA in **Figure S35**. We expand these points in the manuscript.

(Page 11, line 307): "We only assemble RNA:DNA nanostructures from the mRNA and do not target the remaining total RNA background."

(Page 11, line 325): "Translocations of unlabelled total RNA generate non-specific ionic current drops that are readily distinguishable from the highly specific RNA:DNA nanostructure nanopore readout[34], which we exclude from the analysis."

2. Experiment hour ranges from 2 to 8 hours even… under constant DC potential over significant period of time will introduce drift in the current baseline… can the author clarify this?

We appreciate this concern. Despite some drift of the baseline, the signal depth remains almost the same, as well as dwell time, and event area over recording periods of up to 24 hours (*Boskovic, F., 2022, Nat Chem* and *Boskovic, F., 2023, Nat Nanotechnol*). We also published the variability of nanopore observables in *Boskovic, F., 2024, ACS nano*.

We have now added clarification to the Methods section describing baseline monitoring and post-acquisition quality control steps used to ensure signal stability throughout extended recordings. We also include new **Figure S39** where we show baseline monitoring in long measurements.

(Page 18, line 526): "During the nanopore measurements, we monitor that there is no significant ionic current baseline drift that could considerably affect signal depth, dwell time, or event charge deficit (**Figure S39**)."

Figure S39.

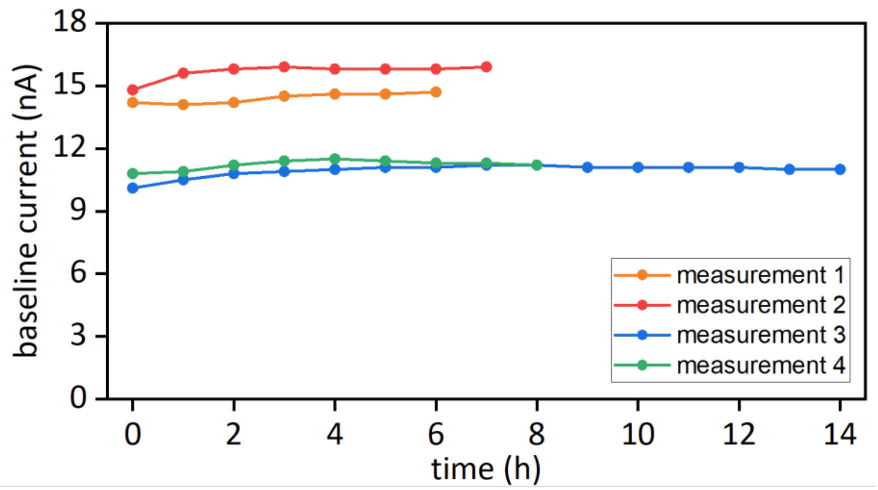

Figure S39. Ionic current baseline for nanopore measurements in 4M LiCl with durations of 6, 7, 8 and 14 hours.

3. Also drying of the reservoir over time? Does the salt concentration slowly increase overtime here due to evaporation… that require further justification.

To minimize LiCl concentration changes due to evaporation, the nanopore reservoirs are within a PDMS device (Bell, N.A.W. et al., Lab on a chip 2013). Once the electrodes are connected to the reservoirs, the chip is sealed with a non-adhesive sealing film (Scotch), which further reduces evaporation. We do not observe significant changes in ionic strength over the duration of the experiments. This information has been added to the Methods section.

(Page 18, line 528): "To minimize LiCl concentration changes caused by evaporation, the nanopore reservoir is effectively sealed within the PDMS device. In addition, a non-adhesive sealing film is placed over the reservoir opening to further reduce evaporation[55]."

4. While multiple nanopore system could be used, the cost of equipment also goes up… I do question whether the author can still resolve the signal trace and spike properly with a lower spec sampling rate of 20 kHz versus 1000 kHz.

Parallelization does not necessarily require proportional increases in hardware cost, as multi-channel acquisitions can be implemented with a 16-channel amplifier (Bell, N.A.W. et al., Lab on a chip 2013). Key barcode features remain resolvable at lower sampling rates (~200 kHz), avoiding the need for MHz-bandwidth instrumentation (Nagpure et al., J. Nanobiotechnol. 2023; N.A.W. Bell et al., Nat. Nanotechnol. 2016). We have clarified this in the revised manuscript:

(Page 12, line 332): "Parallelization of nanopore measurements does not necessarily require a proportional increase in equipment cost, as multiple nanopore acquisitions can be performed in parallel using a single-amplifier architecture[55]. In addition, the barcode labelling features remain resolvable at moderate sampling rates (~200 kHz)[56]."

5. The mRNA detection… to claim it has usage for intracellular detection accurate expansion determination, it should be tested against say 28 CUG expansion versus 34 CUG expansion…

We would like to clarify that we do not claim intracellular detection or precise intracellular expansion counting in the current manuscript. Our conclusions are limited to *ex situ* detection and discrimination of RNA targets with different repeat array lengths, and we have revised the text to make this scope explicit.

(Page 13, line 356): Our conclusions extend to *ex situ* detection and discrimination of RNA targets with different repeat array lengths.

6. The new Figure S16… do the author expect there will be a maximum spike signal drop in this system? i.e. the tool cannot distinguish 51 CUG and 89 CUG?

In addition to the spike depth, spike charge deficit (area) can also be used to discriminate repeat arrays. While the current depth can plateau due to saturation of the sensing volume during nucleic acid translocation, the area continues to scale (Steinbock et al., Electrophoresis 2013), meaning that larger repeat arrays can still be monitored through the current spike area. This point is now stated explicitly in the revised manuscript.

(Page 8, line 224): The current spike depth may approach saturation for larger repeat arrays. However, the integrated signal, which represents the spike charge deficit, continues to increase even when the spike depth plateaus[49]. As a result, reliable discrimination of larger repeat arrays remains possible by analysing the spike area.

Reviewer #2 (Remarks to the Author):

The authors have addressed the majority of my comments. The additions give me greater confidence in the generalizability and accuracy of their approach.

I have one remaining concern about code documentation. I checked the provided URL (https://github.com/maxearle/nas2). Indeed, a README and test data have been added in response to my previous comment; however, there is still insufficient information for me to easily test/use the code. It is possible that this URL is out of date (last edited 3 months ago); so please update it if that is the case.

The README should provide sufficient information for a completely new user to install and run the tool. Here is a resource that provides a good summary and a simple example: https://www.makeareadme.com/

To summarize, at a minimum, the main (top-level) README should include:

- A brief description of the project

- Install instructions (these are present in the current version, but not well formatted)

- A sample run command for the test data

- A list of relevant commands/subcommands/scripts, their uses, parameters and inputs/outputs, including any non-standard formats.

We thank the reviewer for the positive assessment and for the helpful comment on code documentation. We have updated the GitHub repository and substantially revised the top-level README to include (1) a clear project description, (2) formatted installation instructions, (3) an example command using the test dataset, and (4) a summary of the main scripts and their inputs and outputs.

Reviewer #3 (Remarks to the Author):

The revision of the manuscript by Patino-Guillen and collaborators has addressed most of the concerns raised during the initial review.

We are pleased that the revisions have addressed most of the concerns raised by the reviewer.

1. However, the weak point of the study remains the accuracy of hybridization of RNA:DNA hybrid structures in practical applications. In each individual containing variable TR motifs and their corresponding RNA transcripts, the inherent variability in RNA transcription raises mismatches, incomplete hybridization, or disruptions in the reaction in the oligonucleotide panels designed for special TR.

As shown by our cross-hybridization experiments (new **Figure S38**) and multiplexed target identification (new **Figure S37**), these effects do not compromise our assay under the tested conditions. Hybridization thermodynamics further supports this selectivity with the perfectly matched CUG-targeting duplex being favourable than binding to related motifs.

Reply continues in next page

Figure S38.

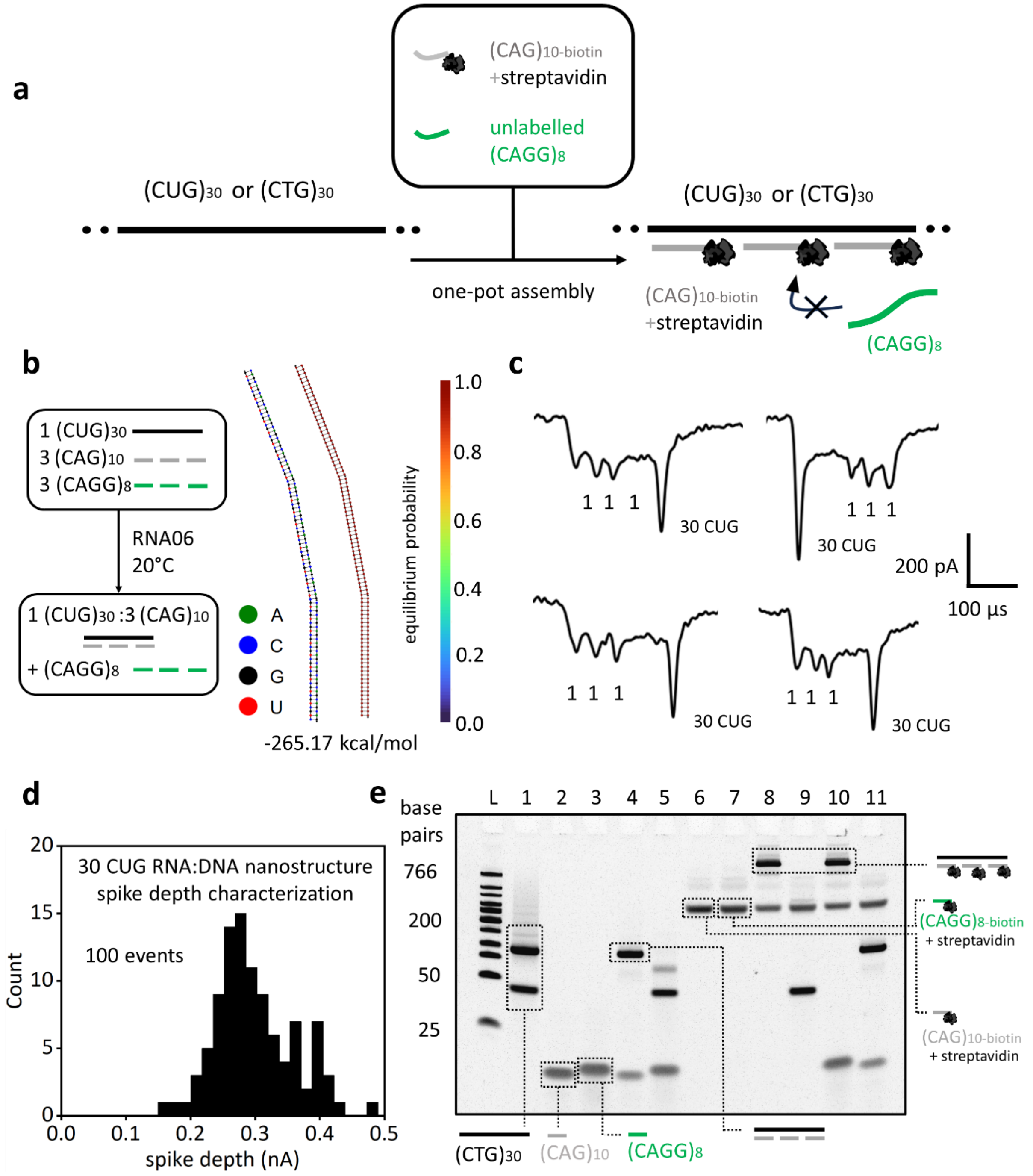

Figure S38. CAG oligonucleotides selectively hybridize to CUG repeats outcompeting CAGG oligonucleotides. **a** We evaluated potential cross-hybridization by testing binding of $(CAG)_{10}$ and $(CAGG)_8$ oligonucleotides to $(CUG)_{30}$ repeats in full-length transcripts with nanopore sensing. We also test hybridization of the oligonucleotides to $(CTG)_{30}$ repeats with native polyacrylamide gel electrophoresis. **b** NUPACK3 with the RNA06 model (including full stacking parameterization)

was used to predict complexes in a mixture of 1:3:3 μM = $(CUG)_{30}$:$(CAG)_{10}$:$(CAGG)_8$ at 20°C. The model predicts 100% of $(CUG)_{30}$ binding to three equivalents of $(CAG)_{10}$. The free energy of the secondary structure is -265.17 kcal/mol. No $(CAGG)_8$ cross-hybridization with $(CUG)_{30}$ is predicted. **c** An RNA:DNA nanostructure was assembled from the transcript containing 30 CUG repeats in the presence of biotinylated $(CAG)_{10}$ oligonucleotides, which recruit streptavidin, together with non-biotinylated $(CAGG)_8$ oligonucleotides, which do not bind streptavidin. Nanopore recordings show translocations with the '111' barcode spikes and an additional spike corresponding to the repeat region. **d** The spike-depth distribution attributed to the repeats indicates streptavidin binding mediated by hybridization of biotinylated $(CAG)_{10}$ oligonucleotides, demonstrating that $(CAG)_{10}$ oligonucleotides outcompete $(CAGG)_8$ oligonucleotides and prevent repeat mislabelling. **e** PAGE further supports selective $(CAG)_{10}$ hybridization specificity outcompeting $(CAGG)_8$ oligonucleotides to $(CTG)_{30}$ repeats. Lane L: Low range DNA ladder. Lane 1: $(CTG)_{30}$. Lane 2: $(CAG)_{10}$. Lane 3: $(CAGG)_8$. Lane 4: $(CTG)_{30}$ + $(CAG)_{10}$. Lane 5: $(CTG)_{30}$ + $(CAGG)_8$. Lane 6: $(CAG)_{10\text{-biotin}}$ + streptavidin. Lane 7: $(CAGG)_{8\text{-biotin}}$ + streptavidin. Lane 8: $(CTG)_{30}$ + $(CAG)_{10\text{-biotin}}$ + streptavidin. Lane 9: $(CTG)_{30}$ + $(CAGG)_{8\text{-biotin}}$ + streptavidin. Lane 10: $(CTG)_{30}$ + $(CAG)_{10\text{-biotin}}$ + $(CAGG)_8$ + streptavidin. Lane 11: $(CTG)_{30}$ + $(CAG)_{10}$ + $(CAGG)_{8\text{-biotin}}$ + streptavidin.

2. This challenge is further compounded when employing multiplexed oligonucleotide panels, as multiple targets increase the likelihood of incorrect binding or incomplete hybridization.

Multiplexing does not pose a major limitation in our approach because we do not hybridize directly to repetitive RNA alone. Instead, we assemble the disease-relevant mRNA transcript, providing a strong initial layer of specificity before engaging the repeat-containing segment. This ensures that closely related repeat-expansion transcripts are excluded at the outset. To clarify this workflow and our transcript characterization, we added a new supplementary figure (**Figure S40**) depicting the steps used for transcript identification and tandem repeat quantitative analysis.

Reply continues in next page

Figure S40.

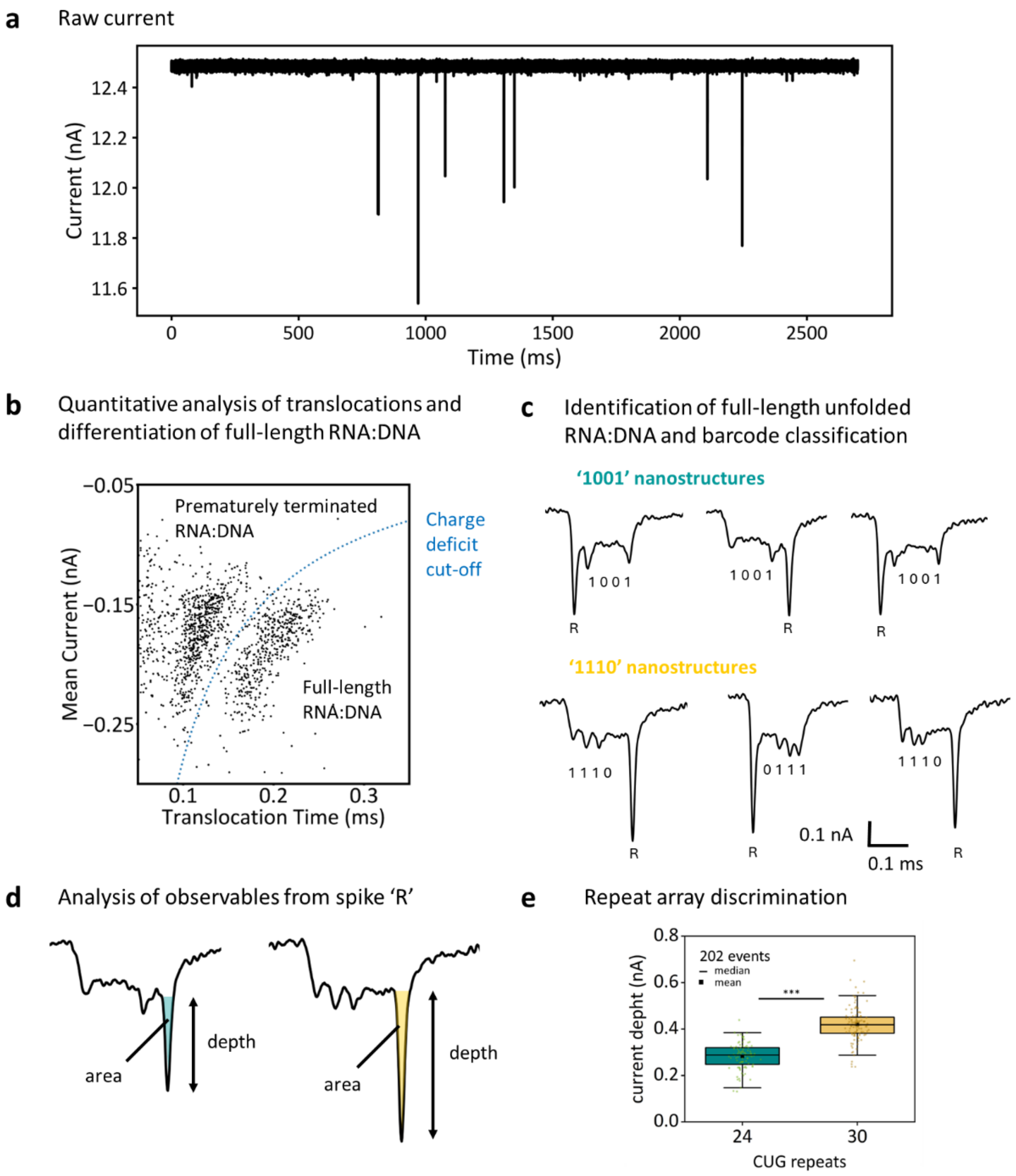

Figure S40. Step-by-step nanopore data analysis of tandem repeat arrays. **a** An example segment of the raw ionic current trace is shown for multiplexed characterization of transcripts containing 24 and 30 tandem repeats. Translocation events are identified using custom LabVIEW software based on simple thresholding. Typical parameters include a translocation duration >0.05 ms, a charge deficit of 10–400 fC, and a mean event current below −100 pA. These criteria enable reliable detection of events corresponding to RNA:DNA nanostructures. **b** Further quantitative analysis of event features (translocation time, charge deficit, mean current) is used to distinguish full-length nanostructures from prematurely terminated or fragmented constructs. **c** Using the nanostructure design and translocation time, unfolded constructs are classified by barcode. In this example, the 24-repeat nanostructure carries a '1001' barcode, whereas the 30-repeat

nanostructure carries a ‘1110’ barcode. **d** Finally, the spike depth and area associated with the tandem repeat region are quantified, **e** enabling discrimination of repeat array length.

Experimentally, we further demonstrate that multiplexing does not compromise specificity. Two RNAs can be targeted in parallel with two oligonucleotide panels in the same mixture (new **Figure S37**). Consistent with our previously reported ≥10^{-5} specificity for closely related 16S rRNA targets (doi: 10.1101/2024.08.15.608086v2), nanopore sensing and PAGE confirm selective nanostructure assembly and repeat hybridization (**Figures S37–S38**). Moreover, repeat expansion disorders are rare, and the likelihood of multiple distinct repeat-expansion targets occurring within a single patient is extremely low.

(Page 17, line 480): The sequence-selective assembly of the nanostructures and repeat hybridization was validated by nanopore sensing and polyacrylamide gel electrophoresis, as shown in **Figures S37** and **S38**.

Figure S37.

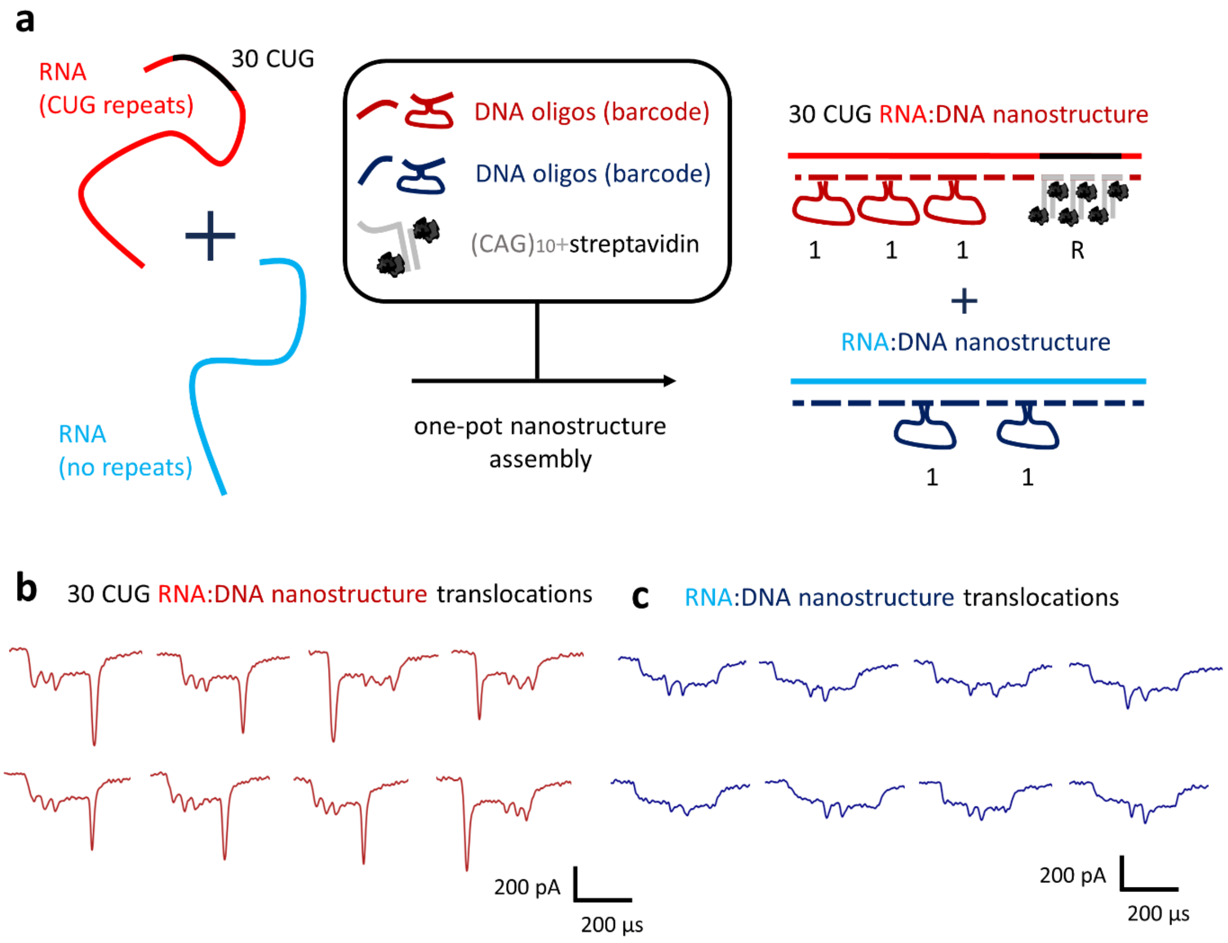

Figure S37. Multiplexed assembly and nanopore characterization of RNA:DNA nanostructures. **a** A 3 kb RNA containing 30 CUG repeats (red) was mixed with a 3.6 kb RNA containing no tandem repeats (blue) in a single reaction tube. DNA oligonucleotides were added to label the CUG-containing RNA with a ‘111’ barcode and the repeat-free RNA with a ‘11’ barcode. Biotinylated

(CAG)10 oligonucleotides were also included to hybridize to the CUG repeats. **b** Nanopore characterization revealed translocations with current spikes attributed to the ‘111’ barcode and an additional spike associated with the CUG repeat region. **c** Nanopore readout using the same nanopore also showed translocations featuring the ‘11’ barcode, corresponding to the transcript without repeats. These results demonstrate multiplexed assembly and nanopore detection of distinct RNA:DNA nanostructures in a single mixture.

3. In your strategy, we have observed two highly similar motif, such as RNA transcription containing both CUG and CCUG at the same time, may cause cross-hybridization in the oligonucleotide panel only designed for CUG or CCUG. To assess hybridization specificity, a mixture of CUG and CCUG RNAs can be applied to an oligonucleotide panel containing immobilized CAG or CAGG single-stranded DNA probes. The ratio of specific to non-specific hybridization can then be determined. If each TR has to be run separately, it is very inefficient and impractical for broader application.

We experimentally addressed the reviewer’s concern about potential cross-hybridization by including oligonucleotide panels targeting both CUG and CCUG repeats while only a CUG-containing RNA was present. The new supplementary figure (**Figure S38**) demonstrates selective hybridization of CAG probes to CUG repeats in full-length transcripts even in the presence of competing CAGG probes designed for CCUG repeats.

Reply continues in next page

Figure S38.

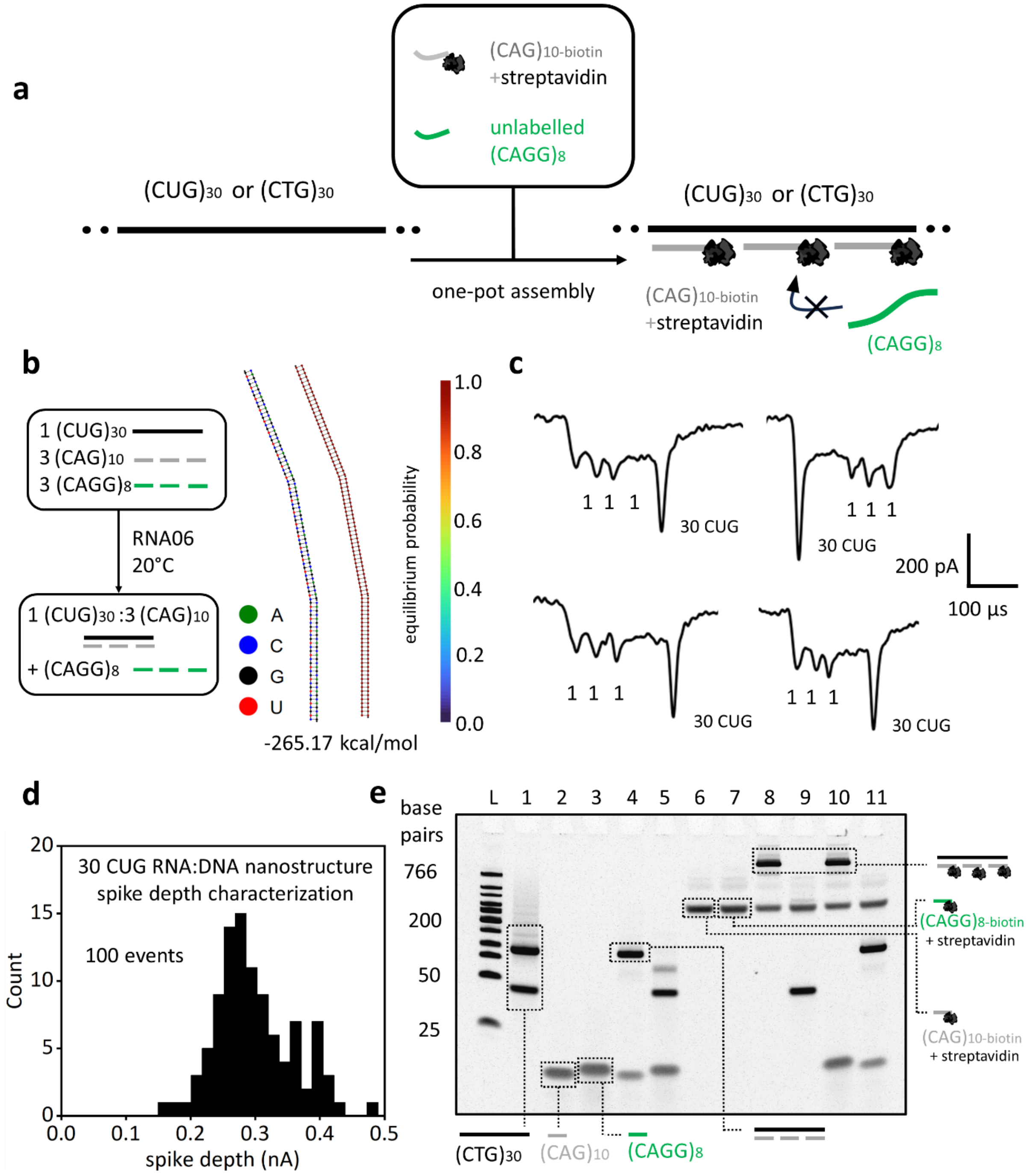

Figure S38. CAG oligonucleotides selectively hybridize to CUG repeats outcompeting CAGG oligonucleotides. **a** We evaluated potential cross-hybridization by testing binding of $(CAG)_{10}$ and $(CAGG)_8$ oligonucleotides to $(CUG)_{30}$ repeats in full-length transcripts with nanopore sensing. We also test hybridization of the oligonucleotides to $(CTG)_{30}$ repeats with native polyacrylamide gel electrophoresis. **b** NUPACK3 with the RNA06 model (including full stacking parameterization)

was used to predict complexes in a mixture of 1:3:3 μM = $(CUG)_{30}$:$(CAG)_{10}$:$(CAGG)_8$ at 20°C. The model predicts 100% of $(CUG)_{30}$ binding to three equivalents of $(CAG)_{10}$. The free energy of the secondary structure is -265.17 kcal/mol. No $(CAGG)_8$ cross-hybridization with $(CUG)_{30}$ is predicted. **c** An RNA:DNA nanostructure was assembled from the transcript containing 30 CUG repeats in the presence of biotinylated $(CAG)_{10}$ oligonucleotides, which recruit streptavidin, together with non-biotinylated $(CAGG)_8$ oligonucleotides, which do not bind streptavidin. Nanopore recordings show translocations with the '111' barcode spikes and an additional spike corresponding to the repeat region. **d** The spike-depth distribution attributed to the repeats indicates streptavidin binding mediated by hybridization of biotinylated $(CAG)_{10}$ oligonucleotides, demonstrating that $(CAG)_{10}$ oligonucleotides outcompete $(CAGG)_8$ oligonucleotides and prevent repeat mislabelling. **e** PAGE further supports selective $(CAG)_{10}$ hybridization specificity outcompeting $(CAGG)_8$ oligonucleotides to $(CUG)_{30}$ repeats. Lane L: Low range DNA ladder. Lane 1: $(CTG)_{30}$. Lane 2: $(CAG)_{10}$. Lane 3: $(CAGG)_8$. Lane 4: $(CTG)_{30}$ + $(CAG)_{10}$. Lane 5: $(CTG)_{30}$ + $(CAGG)_8$. Lane 6: $(CAG)_{10\text{-biotin}}$ + streptavidin. Lane 7: $(CAGG)_{8\text{-biotin}}$ + streptavidin. Lane 8: $(CTG)_{30}$ + $(CAG)_{10\text{-biotin}}$ + streptavidin. Lane 9: $(CTG)_{30}$ + $(CAGG)_{8\text{-biotin}}$ + streptavidin. Lane 10: $(CTG)_{30}$ + $(CAG)_{10\text{-biotin}}$ + $(CAGG)_8$ + streptavidin. Lane 11: $(CTG)_{30}$ + $(CAG)_{10}$ + $(CAGG)_{8\text{-biotin}}$ + streptavidin.

Response letter to the reviewers for the manuscript:

Quantification of disease-associated RNA tandem repeats by nanopore sensing

Patiño-Guillén, G.[1], Pešović, J.[2] , Panić, M.[3], Earle, M.[1], Ninković, A.[2], Petrușca, S.[1], Savić-Pavićević, D.[2,*], Keyser, U.F.[1,*] , Bošković, F.[1,*]

[1]Cavendish Laboratory, University of Cambridge, Cambridge, UK

[2]University of Belgrade – Faculty of Biology, Centre for Human Molecular Genetics, Belgrade, Serbia

[3]Institute of Virology, Vaccines and Sera "Torlak", Belgrade, Serbia

*Corresponding authors: fnb24@cam.ac.uk, ufk20@cam.ac.uk, duska@bio.bg.ac.rs

We are grateful to the reviewers for the time devoted to assessing our manuscript. Below, we provide a response to each point raised. The reviewers' points are in italic, and our responses are in regular font. The modifications implemented in the manuscript are highlighted in red.

REVIEWER COMMENTS

Reviewer #1 (Remarks to the Author):

Supporting figure capture rate is captioned as capture rate (1 / hr nM). This requires further explanation, how does one translate that back to molecules per hour?

To translate to molecules per hour, the capture rate (1 / hr nM) must be multiplied by the concentration of RNA:DNA nanostructure (nM) used during the nanopore measurement. We have added a brief statement in the manuscript indicating how to derive the molecules per hour.

"The reported capture rate is normalized to analyte concentration, so molecules captured per hour are obtained by multiplying the value in h^{-1} nM^{-1} by the RNA:DNA nanostructure concentration used in the nanopore measurement."

Accordinig to Bell et al.'s paper that author mentioned, the maximum sampling rate is 20 kHz if all 16 channels are being used, as stated in the said paper. The author's argument is only valid if each channel of the single amplifier system (e.g. the 16 channel one) operates at 20 kHz. Say for a 0.3 ms translocation signal that the RNA:DNA structure generates, sampling at 100 kHz (which is the lower end and minimum accepted sampling rate in solid-state nanopore system most lab will have to use), you will have 30 data points, if you sample at 20 kHz, you have 6 data points.
What I tried to ask is that if your entire trace has 6 data points, can you still resolve the e,g, 1001 + R spike as you demonstrated throughout (simply put, it all these signals that the paper presented have 6 points, would you still be able to differentiate 1001+R spike signal? Next, the signal attenuation brought by the usage of low-pass filter as shown cleanly in this paper: https://pubs.acs.org/doi/10.1021/ac901877z, the spike signal at such low sampling frequency will be further attenuated to become almost like a baseline. These are realistic conditions that I do not think the author still able to address, and this technology still require custom solutions on high-end electronics, if the author wants to do real time diagnostic in places like hospital with this technology.

We agree that six sampled points per event would not be sufficient to resolve the internal spike structure shown here, and anti-aliasing at 20 kHz would further suppress these short features. Accordingly, our results should not be interpreted as claiming immediate compatibility with such low-bandwidth acquisition. Translation to those conditions would require redesign of the readout, for example by slowing translocation or using broader event features rather than fine substructure. Hardware simplification for "cheaper instrument" lies outside the scope of the present study, especially since most genetic diagnostics are conducted in centralized laboratories equipped with specialized instruments. We added sentence to mention these as a potential frontier:

"In the future, RNA:DNA nanostructures could be engineered to produce simpler and more slowly varying signals that may be readable with lower-cost electronics and machine learning[57,58], similar to Oxford Nanopore Technologies nanopore sequencing platforms."

Reviewer #2 (Remarks to the Author):

The authors have addressed all my comments. Thank you!

We thank the reviewer for the comments.

Reviewer #3 (Remarks to the Author):

The author has adequately addressed my comments. The results are clearly presented and well-supported by experimental data and prior research. Overall, this work is suitable for publication.

We thank the reviewer for the comments.
